# Structural and transcriptional analysis of plant genes encoding the bifunctional lysine ketoglutarate reductase saccharopine dehydrogenase enzyme

**DOI:** 10.1186/1471-2229-10-113

**Published:** 2010-06-16

**Authors:** Olin D Anderson, Devin Coleman-Derr, Yong Q Gu, Sekou Heath

**Affiliations:** 1 Genomics and Gene Discovery Research Unit, Western Regional Research Center, USDA-ARS, 800 Buchanan Street, Albany, CA 94710, USA; 2 Department of Plant Sciences, University of California, Berkeley, CA 94720, USA; 3 783 Euclid Avenue, Berkeley, CA 94708, USA

## Abstract

**Background:**

Among the dietary essential amino acids, the most severely limiting in the cereals is lysine. Since cereals make up half of the human diet, lysine limitation has quality/nutritional consequences. The breakdown of lysine is controlled mainly by the catabolic bifunctional enzyme lysine ketoglutarate reductase - saccharopine dehydrogenase (LKR/SDH). The LKR/SDH gene has been reported to produce transcripts for the bifunctional enzyme and separate monofunctional transcripts. In addition to lysine metabolism, this gene has been implicated in a number of metabolic and developmental pathways, which along with its production of multiple transcript types and complex exon/intron structure suggest an important node in plant metabolism. Understanding more about the LKR/SDH gene is thus interesting both from applied standpoint and for basic plant metabolism.

**Results:**

The current report describes a wheat genomic fragment containing an LKR/SDH gene and adjacent genes. The wheat LKR/SDH genomic segment was found to originate from the A-genome of wheat, and EST analysis indicates all three LKR/SDH genes in hexaploid wheat are transcriptionally active. A comparison of a set of plant LKR/SDH genes suggests regions of greater sequence conservation likely related to critical enzymatic functions and metabolic controls. Although most plants contain only a single LKR/SDH gene per genome, poplar contains at least two functional bifunctional genes in addition to a monofunctional LKR gene. Analysis of ESTs finds evidence for monofunctional LKR transcripts in switchgrass, and monofunctional SDH transcripts in wheat, *Brachypodium*, and poplar.

**Conclusions:**

The analysis of a wheat LKR/SDH gene and comparative structural and functional analyses among available plant genes provides new information on this important gene. Both the structure of the LKR/SDH gene and the immediately adjacent genes show lineage-specific differences between monocots and dicots, and findings suggest variation in activity of LKR/SDH genes among plants. Although most plant genomes seem to contain a single conserved LKR/SDH gene per genome, poplar possesses multiple contiguous genes. A preponderance of SDH transcripts suggests the LKR region may be more rate-limiting. Only switchgrass has EST evidence for LKR monofunctional transcripts. Evidence for monofunctional SDH transcripts shows a novel intron in wheat, *Brachypodium*, and poplar.

## Background

Monogastric mammals, which include humans, depend on external dietary sources for half of the amino acids needed for protein synthesis. The aspartate-family pathway controls synthesis of the essential amino acids lysine, threonine, and methionine, with lysine feedback-inhibition and rates of lysine degradation being factors in this important pathway. Among the essential amino acids, lysine is the most severely limiting in the cereals - crops that make up half of the human diet [1]. In contrast to animals, plants synthesize lysine and have evolved complex metabolic pathways to maintain lysine levels [2]. To understand lysine metabolism, a thorough understanding of all aspects of these pathways is necessary. For the catabolic portion of lysine metabolism, the bifunctional enzyme lysine ketoglutarate reductase saccharopine dehydrogenase (LKR/SDH; synonym α-aminoadipic-δ-semialdehehyde synthase) converts lysine to glutamate and α-aminoadipic acid via a 2-step pathway; i.e., the LKR activity (E.C. 1.5.1.8) catalyzes the formation of saccharopine from lysine and α-ketoglutarate (2-oxoglutarate), and the SDH activity (E.C. 1.5.1.9) processes the saccharopine into glutamate and an α-aminoadipic-δ-semialdehehyde which is further catabolized to two glutamates [2,3]. In both plants and animals, the *LKR/SDH *gene encodes an open reading frame composed of fused LKR and SDH domains - compared to yeast and fungi where the LKR and SDH activities are encoded by separate genes [4,5]. In plant *LKR/SDH *genes, there is a linker, or interdomain, sequence not present in animals that separates the LKR and SDH encoding domains - leading to speculation that there are controls and functions unique to plants [6]. Both LKR/SDH and monofunctional SDH mRNAs have been detected in mouse [7]. Similarly in plants, an *Arabidopsis *SDH mRNA is reported that initiates transcription inside the 3' sequence of the interdomain [8], and a cotton LKR mRNA is reported that terminates at the 5' junction area of the linker [9]. One question in the latter report was that the 3' noncoding sequence is not present in the *LKR/SDH *gene - leaving the origin of this sequence uncertain but attributed to a possible trans-splicing event. The function of such mono-functional mRNAs is not clear, but the SDH mRNA and protein levels were consistently higher than the LKR/SDH mRNA and protein levels in *Arabidopsis *tissues - leading to the proposal that the LKR activity was the rate limiting step and that the relative SDH excess assured rapid flux through the pathway for LKR/SDH [10].

The exact site of activity of LKR/SDH is considered to be the mitochondria [11], but evidence is not clear. The LKR/SDH enzyme has been localized to the cytosol in plants [6,12], while lysine-α-ketoglutarate reductase and saccharopine dehydrogenase enzymatic activities were located only in the mitochondrial matrix in animal livers [13,14]. Possible roles in transcription regulation include evidence of LKR/SDH being a co-factor involved in hormone-mediated transcription through regulation of H3 and H4 histone methylation [15] and the *LKR/SDH *gene is reported to be regulated by the *Opaque2*-type transcription factors that also control the expression of at least some classes of cereal seed proteins [6]. In addition to a direct role in lysine metabolism, LKR/SDH has been reported to be regulated by a number of environmental and metabolic influences including osmotic balance, hormome levels, and salt and water stresses [10,16,17]. Suggestive evidence for more complex regulatory roles for LKR/SDH are that expression is enhanced in developing seeds of cereals and floral tissues known to contain limited amounts of lysine, and analyses suggesting LKR/SDH expression is not highly coordinated with other catabolic enzymes [8]. Similarly, the finding of multiple transcripts from the same gene (encoding mono- and bifunctional enzymes) and a coding region composed of 25 exons in a dicot and 26 exons in a monocot [12] suggests complex regulation and roles in plant metabolism and development [2,10].

The importance of lysine to animal/human nutrition and the role of LKR/SDH in lysine catabolism has lead to several approaches to increase plant seed lysine. These approaches include increasing seed lysine by transformation with feedback-insenstive versions of lysine anabolic genes [18], down-regulating the *LKR/SDH *gene [19], a combination of those two approaches [20], transgenic expression of a foreign protein high in lysine [21], and reducing synthesis of lysine-poor seed proteins [22].

Plant LKR/SDH genomic sequences have been formally reported only for *Arabidopsis *[8,23] and maize [6]. A comparison of these dicot and monocot genes found high conservation in exon size and sequence, with the maize gene having one additional exon in the 5' region [12]. The dicot and moncot intron sequences have diverged completely and the maize introns are generally larger - from start to stop codons the maize *LKR/SDH *gene spans 9515 bp while the *Arabidopsis *gene spans 5590 bp. Additional plant LKR/SDH sequences are available (rice, poplar, grape, etc.), but have not been comparatively analyzed. The Triticeae crops (wheat, barley, rye, triticale) are, as a group, the largest direct fraction of the human diet worldwide, but no *LKR/SDH *gene has been reported for this important crop group.

The current report describes a BAC clone of a wheat genomic fragment containing an *LKR/SDH *gene, determines genome assignments of the BAC and EST contigs in hexaploid wheat, and compares relative homoeologue expression among the three hexaploid wheat genomes. Also described are a comparative analysis of a set of plant *LKR/SDH *genes including variant structures in the poplar and grape genomes. Wheat and other plant LKR/SDH ESTs are analyzed to determine splicing sites and evidence for alternative splicing. This analysis also finds EST evidence for both monofunctional LKR and SDH transcripts.

## Results and Discussion

### Wheat LKR genomic region

To isolate a wheat *LKR/SDH *gene, a durum wheat (*Triticum turgidum*) tetraploid 5× BAC library was screened. Six BACs were positive for LKR/SDH sequences and formed two contigs of four and two BACS respectively, as seen from Southern analysis and BAC fingerprinting (not shown). Each contig contained single LKR/SDH sequences - suggesting that there are single *LKR/SDH *genes in each of the wheat A and B genomes of tetraploid wheat. BAC 0006M07 was chosen for sequencing as having the *LKR/SDH *gene relatively centrally located in the BAC. This BAC was sequenced and found to be 161,506 bp in length. The sequence can be found as Genbank accession GU182251.

**Figure 1:**
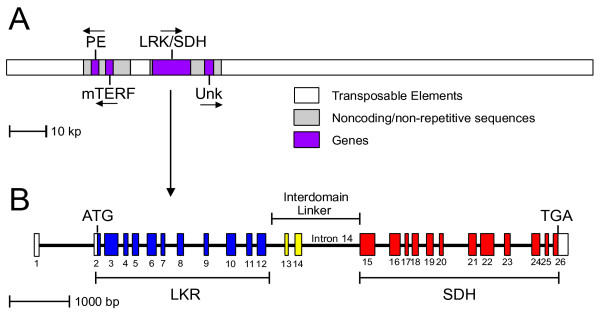
**Organization of an *LKR/SDH *gene segment of the wheat genome**. (A) Diagram of the wheat LKR/SDH genomic region spanned by BAC clone 0006M07. Genes are identified by purple boxes and regions of transposable elements by white boxes. Sequences of non-coding and non-repetitive type are indicated by grey boxes. Arrows indicate direction of transcription. (B) The *LKR/SDH *gene region is expanded to show exon/intron organization. Exons are indicated by numbered boxes and introns by intervening lines. The LKR domain exon boxes are blue and the SDH boxes are red. Two yellow exons are the proposed interdomain coding region of the full-length LKR/SDH protein. Start and stop codon positions of the full-length coding region are marked.

The annotation of this region of the wheat genome found three known genes and one unknown gene as shown in Figure 1A; i.e., genes for a pectinesterase (PE), a mitochondrial termination factor (mTERF), *LKR/SDH*, and an unknown gene. These four genes are clustered within about 43,000 bp composed of two pairs of gene sequences (purple boxes in Figure 1A) and non-coding and non-repetitive sequences (grey boxes) that include gene promoters. These two gene pairs are separated by a short region of transposable elements. The remainder of the 161,506 bp BAC sequence is composed of nested transposable elements of various classes (white boxes). This organization is consistent with previous reports that the wheat genome is composed of small "islands" of 1-4 genes separated by regions of transposable elements [24-27].

### Structure of the a wheat LKR/SDH gene and protein

Analysis of the wheat *LKR/SDH *gene sequence indicates the structure shown in Figure 1B. Consensus exon/intron boundaries were determined using wheat EST sequences aligned to the genomic sequence. At least one wheat EST exists that overlaps all the coding sequence except for the region around exon 10 where maize and rice LKR/SDH coding sequences were used to estimate exon/intron boundaries. In regions with only 1-2 wheat ESTs, exon/intron boundaries matched rice and maize sequences in all cases. Similar to the previously reported maize *LKR/SDH *gene structure [10], the wheat *LKR/SDH *gene is comprised of 26 exons and 25 introns. The intron borders matched the canonical plant intron borders (GT...AG) for all 26 introns. The 5' portion of the sequence encodes the LKR activity of the bifunctional enzyme, which is encoded by eleven exons (blue boxes in Figure 1B); the 3' part of the sequence encodes for SDH activity and contains twelve exons (red boxes). The two regions are separated by an interdomain region composed of two exons (yellow boxes) and three introns, one of which (intron 14) is the longest intron in the *LKR/SDH *gene (1122 bp). This intron may include 5'-UTR/promoter sequences for monofunctional SDH transcripts (see below).

**Figure 2:**
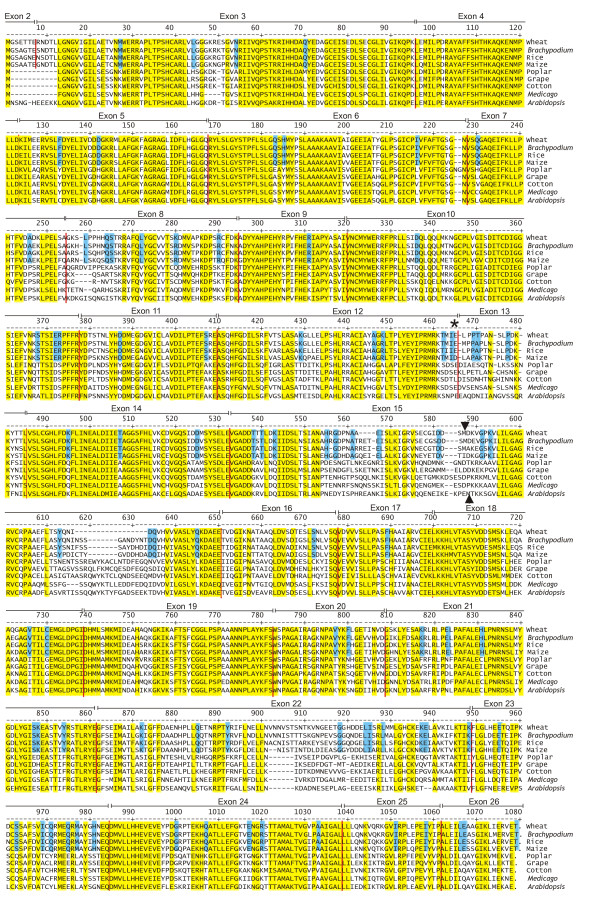
**LKR/SDH amino acid sequences**. The derived amino acid sequence of a wheat LKR/SDH protein is shown and compared to sequences derived from DNA sequences of other plants. Amino acid positions with no more than one difference among all plants are shaded yellow. Amino acids unique to monocots are shaded blue. Red lines indicate exon boundaries. Arrowheads indicate predicted start positions of monofunctional SDH transcripts for wheat (above sequences) and *Arabidopsis *(below sequences). An asterisk marks the approximately position ending the monofunctional LKR transcript. Exon numbers above the sequences indicate monocot exon numbers - dicot numbering is one less since exons 1 and 2 are fused in dicots. The poplar sequence is derived from poplar gene 1 as described below.

The coding sequence from the wheat *LKR/SDH *gene is used to derive the complete bifunctional amino acid sequence and is shown in Figure 2 along with indications of exon boundaries (exon 1 is entirely 5' UTR sequence). A similar analysis was carried out for available sequences from *Brachypodium*, cotton, grape, *Medicago*, poplar, and rice. These seven sequences are compared to the previously reported sequences from *Arabidopsis *and maize and the nine derived amino acid sequences are aligned in Figure 2. Highlighted are conserved amino acid positions (no more than one difference) on all analyzed plants (yellow) and positions unique to monocots (blue). Exon/introns positions are conserved among all plants analyzed with an exception that exons 2 and 3 in monocots form a single exon in dicots as previously noted [10]. Exons are indicated for the monocots (dicot exon numbers are one less since exons 1 and 2 are fused in dicots, i.e., monocot exon 15 is homologous to dicot exon 14). Although much of the sequence is conserved among all plants, several portions are not and are characterized by both residue differences and sequence length variation. Examples of major differences between monocots and dicots include the beginning of exon 8, the junctions of exons 12 and 13, exon 15, and exon 22. The exons 12 and 13 junction where differences in transcription termination suggest monofunctional LKR transcripts (more below). Exons 8 and 22 are in the central portion of the LKR and SDH domains, respectively. Exon 15 is both at the beginning of the SDH domain and one of the largest exons. Exon 14 is one of the most conserved exons, but is part of the interdomain region and not the LKR and SDH enzymatic domains, suggesting conservation of function not yet understood. The wheat LKR/SDH polypeptide is similar in length to the other grass polypeptides except at positions starting at 563 and 617 in exon 15 of Figure 2 - the wheat sequence is three and ten amino acid residues shorter than in the other grasses, respectively. The functional significance of such differences is not known, but may relate to regions of limited functional significance.

**Figure 3:**
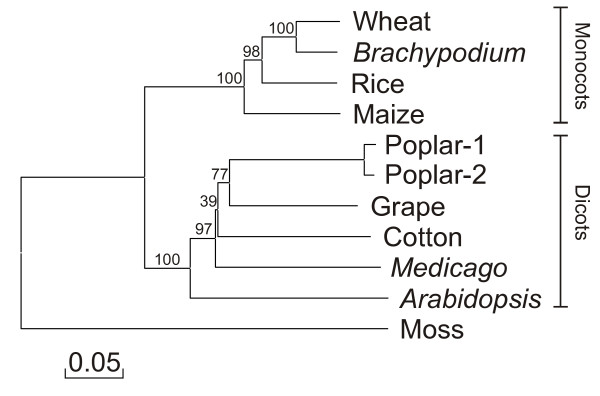
**Phylogenetic analysis of LKR/SDH proteins**. LKR/SDH amino acid sequences were used to generate a phylogenetic tree using Clustal W and described in Methods. Length of branches represent the number of amino acid substitutions per site. The percentage of replicate trees in which the associated sequences clustered in the bootstrap test are shown next to the branch points. Clusters of monocot and dicot sequences are labeled.

As suggested by the blue shading in the amino acid alignment, the *LKR/SDH *amino acid sequences from dicots and monocots form two distinct branches on phylogenetic analysis as shown more clearly in Figure 3. A pair-wise distance table is given in Additional File 1. The closest related sequence to wheat is from *Brachypodium*, consistent with previous reports from BAC-end sequence analyses [28] and from taxonomic placement of *Brachypodium *in the Brachypodieae tribe sibling to the Triticeae tribe (that includes wheat and barley) - both tribes are members of the Pooideae subfamily (ncbi.nlm.nih.gov/Taxonomy). The tree in Figure 3 also shows that the only two previously described *LKR/SDH *sequences, from *Arabidopsis *and maize (which are considered models for their respective groups), are each most distantly related to the other plant proteins within their respective groups.

The coding regions of available LKR/SDH genes were also compared and formed a phylogenetic tree of the same form as in Figure 3 (not shown). A comparison of intron sequences found no significant conservation of intron sequences for available LKR/SDH sequences within both dicots and monocots - with the exception of the *Brachypodium*/wheat comparison where significant conservation is evident (Additional File 2). Further sequencing of LKR/SDH, and other genes from a larger panel of more closely related plant genera and species is needed to understand patterns of plant intron sequence divergence.

### Chromosome and genome location of the wheat LKR/SDH gene

Southern analysis of hexaploid wheat found that wheat EST BE606591 hybridized to three genomic fragments [29]http://wheat.pw.usda.gov/wEST. Two of the LKR/SDH-encoding fragments were mapped to the 6AL and 6BL chromosome arms but a third fragment could not be accurately mapped or assignment made to the 6DL chromosome (see Additional File 3). The same analysis localized the gene to the region of 0.4 to 0.55 of the wheat consensus group 6 chromosome long arm. The wheat group 6 chromosomes have most homology to rice chromosome 2 [30] - consistent with the wheat *LKR/SDH *gene on the group 6 chromosomes since the rice *LKR/SDH *gene sequence is found on rice chromosome 2 http://www.ncbi.nlm.nih.gov.

**Figure 4:**
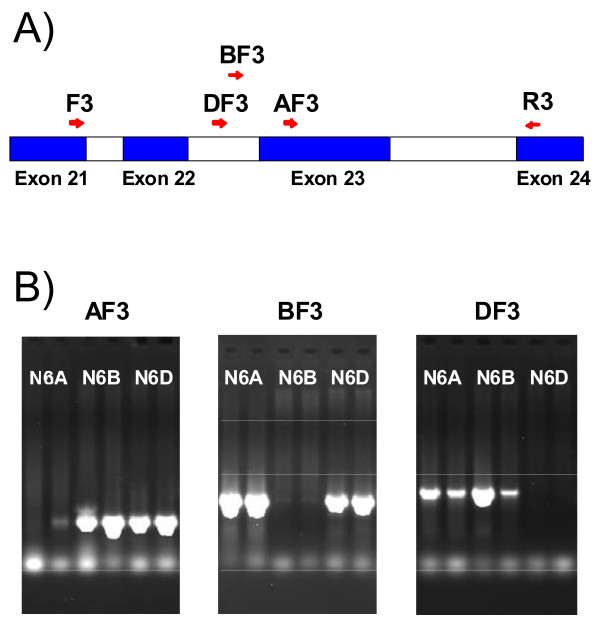
**Genome-specific LKR/SDH primers**. Common priming sites for all three genomes are F3 and R3. Genome-specific priming sites were developed in intron 22 for the B- and D-genomes (BF3 and DF3, respectively) and in exon 23 for the A-genome (AF3). (B) PCR products generated with each genome-specific LKR/SDH primer plus common primer R3 from genomic DNA of hexaploid wheat cv Chinese Spring group 6 chromosome nullisomic-tetrasomic genetic lines (N6A = nulli6A, N6B = nulli6B, N6D = nulli6D). Two amounts of sample were loaded for each DNA.

The wheat *LKR/SDH *gene sequence of BAC 0006M07 was compared to rice and conserved primers were designed and used to amplify DNA from diploid ancestors of the hexaploid wheat (*T. aestivum*; A, B, and D genomes) and tetraploid wheats (*T. turgidum*; A and B genomes). Amplified fragments were sequenced and used to design A-, B-, and D-genome specific primers (see Materials). Genome-specific primer pairs are shown to amplify from specific genomes using three wheat nullisomic-tetrasomic genetic stocks - each stock missing one of the three group 6 wheat chromosomes (Figure 4). These genome-specific primer pairs were used to determine the genome origin of the wheat BAC 0006M07. Results showed that primer pair AF3 (A-genome specific) and R3 (universal for all wheat genomes) amplified the expected fragment size from BAC 0006M07 and DNAs containing the A-genome (nulli6B-tetra6D, tetraploid cultivar Langdon), but not from DNA missing the A-genome (nulli6A-tetra6D) - establishing that BAC 0006M07 originated from the wheat A-genome (Additional File 4).

The previous results supported *LKR/SDH *genes exist in each of the hexaploid wheat's A-, B-, and D-genomes. To determine if all three genes were actively transcribed, wheat LKR/SDH ESTs were identified (Additional File 5). These ESTs assembled into three contigs, one of which (contig 1) is identical over its 1574 bp to the sequenced BAC LKR/SDH coding region (not shown). The relationship of the three contigs to the same region in the BAC sequence is shown in Additional File 6. Contig 1 also contains all five *T. monococcum *(diploid grass related to the wheat A-genome donor) LKR/SDH ESTs, therefore confirming contig 1 and the BAC as originating from the A-genome. Contigs 1 and 2 contain, respectively, 7 and 17 ESTs from tetraploid wheats (A- and B-genomes). Contig 3 contains no tetraploid or *T. monococcum *ESTs. Therefore, contig 2 should represent the B-genome and contig 3 the D-genome. In support of these assignments, the sequences of contigs 1 and 3 are closer to each other than to contig 2 (Additional File 7), consistent with the previous reports that the A and D common ancestor diverged from the B-genome ancestor [27]. Finally, the sequences amplified from specific genomes matched the three contigs and confirmed the genome assignments (not shown).

When the ESTs for the three hexaploid wheat LKR/SDH EST contigs are tallied, the distribution by genome for the A-, B-, and D-genomes is 54, 47, and 35 ESTs, respectively. A Chi-square goodness-of-fit test for departure from expected values yields P = 0.13. Thus, the number of ESTs from the three homoeologs is not considered statistically significantly different from the expected numbers. Further, more global, analyses are needed to understand whether differential homoeologue transcription has a role in polyploid plants.

### Structure and expression of genes adjacent to the LKR/SDH locus

Three other genes are found near the *LKR/SDH *gene in this study (Figure 1). The first gene is for a pectinesterase (PE; a.k.a. pectin methylesterase). This class of enzymes catalyses the demethylesterification of cell wall polygalacturonans and produces de-esterified acidic pectins and methanol [31]. The plant pectinesterases comprise a large family of enzymes with roles in a wide range of plant cell activities including cell adhesion, cell elongation, organelle formation, ribosome binding, and plant defense [31-33]. The pectinesterase gene in BAC 0006M07 contains one intron of 470 bp (not shown). No ESTs are found that exactly match the BAC A-genome pectinesterase gene, but Additional File 7 shows four similar wheat ESTs (BQ806129, CA717792, CJ525781, CJ634274) with 93-96% sequences matches to the BAC pectinesterase gene. These four ESTs are likely from one of the orthologous PE genes in either the B- or D-genomes. In addition, the best BLASTn match of these ESTs and the BAC PE sequence is to the rice pectinesterase gene adjacent to the *LKR/SDH *gene in the rice genome (not shown).

**Figure 5:**
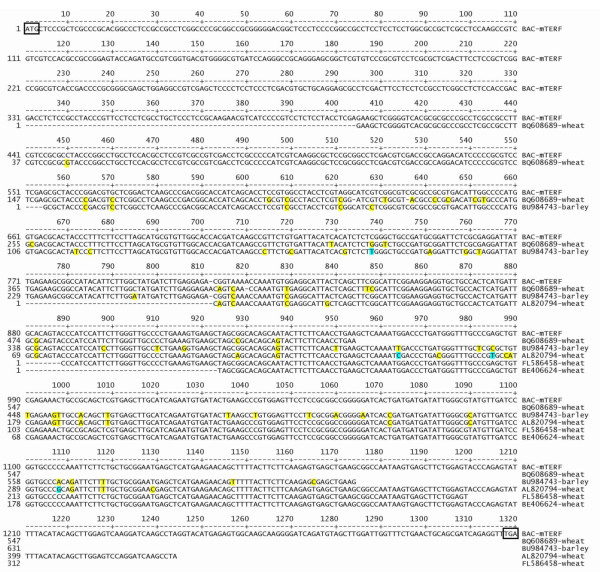
**ESTs matching BAC mTERF gene**. Wheat and barley ESTs matching the BAC mTERF gene are aligned with the mTERF coding sequence. Start and stop codons are boxed. Sequence differences to the BAC are shaded in yellow and blue.

The second additional gene encodes a mitochondrial termination factor (mTERF). These genes encode a protein family involved in the transcriptional regulation of the mitochondrial genome. Mitochondrial DNA is transcribed as polycistrons that include RNA for rRNA, tRNA, and mRNAs. A preponderance of rRNAs is achieved by mTERFs promoting transcription termination at the 3' end of the rRNA region [34] and pausing transcription at other sites in the mitochondrial genome [35]. An mTERF gene is found in the BAC sequence between the pectinesterase and LKR/SDH genes at about 28,000 bp (Figure 1). The gene contains no introns and has an intact mTERF reading frame that encodes a mTERF highly similar to mTERF proteins reported for other plants - an amino acid alignment is shown in Additional File 8 for rice and maize. The wheat BAC mTERF gene is also expressed since several good matches to wheat ESTs exist (Figure 5) including wheat ESTs BE406624 and FL586458 which are exact matches over their 365 and 312 bp lengths, respectively, and are therefore assigned to the A-genome. Wheat ESTs BQ608689 and AL820794 have DNA sequences that are 94% matching the wheat A-genome LKR/SDH sequence, and are likely from the B- and/or D-genome orthologous genes. A single barley EST is also a 94% DNA sequence match to the wheat A-genome LKR/SDH coding sequence. The best rice genomic similarity to the BAC mTERF and matching ESTs is to an mTERF gene adjacent to the rice *LKR/SDH *gene.

Finally, immediately 3' to the *LKR/SDH *gene is an apparent gene of unknown function. Similar genomic or EST sequences are found only in the Triticeae, but a unique 17 out of 18 bp sequence is found in the same relative position 3' to the *LKR/SDH *gene in rice. Although no wheat ESTs exactly match this unknown gene, a similar region is apparently transcribed in barley since two barley ESTs (BM099304 and BM372530) are close matches (Additional File 9). The alignment of the two barley ESTs to the wheat genomic region shows seven gaps with canonical intro/exon junctions at 14 of 15 positions. When the apparent exons are spliced together and the resulting DNA and derived amino acid sequences are used as queries in database searches, no significant match is found to any DNA (best match e = 0.1) or protein sequences (best match e = 0.28). The two barley ESTs are from pistil and embryo sac, respectively. These two tissues have not been commonly sampled for ESTs - which could account for the sequence not appearing in other plant EST collections. If this sequence is found only in Triticeae, then the sequence must have arisen after separation of the Triticeae from other grasses. Thus, although the intron/exon structure and ESTs argue for a functional gene, this remains to be further established.

### Comparison to other genomes

**Figure 6:**
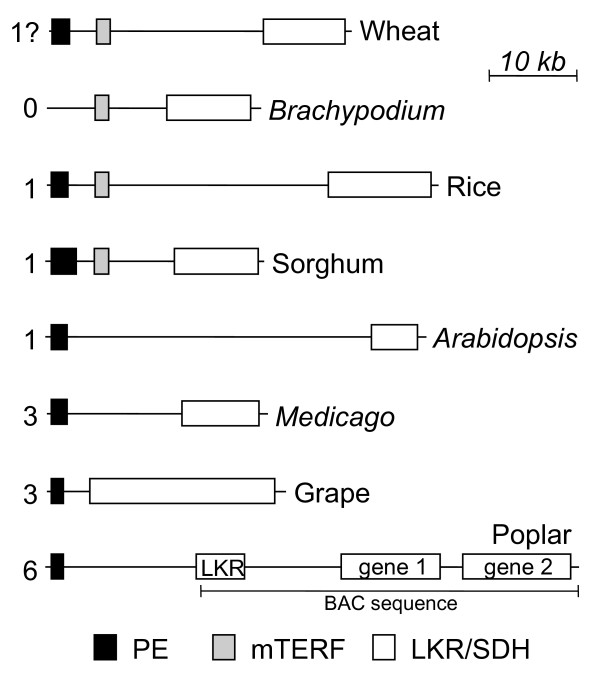
**Compare LKR/SDH genome regions**. LKR/SDH genomic regions of eight plants are compared for the relative location of three genes, i.e., *LKR/SDH*, mTERF, and PE. *LKR/SDH *genes = white boxes. mTERF genes = grey boxes. PE genes = black boxes. The numbers at the left indicated the total number of PE genes found 5' to the LKR/SDH genes in available genomic sequences. No additional wheat genomic sequence is available as indicated by the question mark. Gene lengths and spacing are drawn to scale. Gene lengths are the sum of exons plus introns. The three poplar genes are labeled LKR (monofunctional LKR) and genes 1 and 2 for the two full-length *LKR/SDH *genes. The poplar LKR/SDH genes are from BAC AC209229 and the PE genes from the genome assembly.

In addition to comparing the LKR/SDH derived protein sequences (Figure 2), the region of the wheat genome represented within BAC 0006M07 was compared to other available plant genomic sequences, either from complete genomes in the cases of *Arabidopsis*, *Brachypodium*, *Medicago*, rice, and sorghum, or from BAC sequences containing LKR sequences and some flanking DNA as in the cases of grape and poplar (Figure 6). In seven of the eight species, the data supports a single *LKR/SDH *gene per genome. The exception was for poplar, where poplar BAC AC209229 (Genbank) contains two full-length copies of the *LKR/SDH *gene plus a third gene encoding only the LKR portion with a 5' LKR sequence truncation at the end of the BAC sequence. In contrast, searching the *Poplar trichocarpa *genome sequence http://genome.jgi-psf.org found one apparently full-length *LKR/SDH *gene flanked by two partial genes containing either a fragmented LKR region or a fragmented SDH region, respectively. For further discussion, the poplar genes are referred to as the LKR gene 1 and gene 2. Several observations argue against the poplar BAC sequence being an artifact and that the BAC sequence assembly is more accurate than the current version of the *P. trichocarpa *genome sequence. When aligned, the three poplar gene sequences show more conservation of exon sequences than intron sequences which have major divergences (not shown), and the coding sequences all have distinct differences. Although the few poplar LKR/SDH ESTs are from 3-4 different species of *Populus (P. nigra,P. trichocarpa, P. tremula, and a P. tremula x P. tremuloides *cross), the two complete LKR/SDH genes have distinctive 3' UTRs with matching ESTs (Additional File 10) that indicates ESTs originating from both genes (gene 1 matches ESTs CV242527, DV242515, DB907693, DB900296; gene 2 matches ESTs DB899866, CK105181, CK095239, BI126461). The most likely cause of the discrepancy between the BAC and the genomic assembly is the three tandem *LKR/SDH *gene sequences led to errors in the shotgun genome assembly, although not ruled out are differences between *P. trichocarpa *germplasms.

The poplar monofunctional LKR gene in ends at about 200 bp 3' to the end of exon 11 when compared to the full-length genes (not shown). In addition, the sequence has a frameshift in exon 7 (not shown) caused by a TC to TCTC difference in sequence compared to the two full-length genes. Only five ESTs are available for the 5' end of the poplar LKR sequences and none match the monofunctional LKR gene sufficient to suggest any transcripts from that gene. However, these are too few ESTs to rule out gene activity, and the apparent frameshift would need to be confirmed.

Figure 6 compares adjacent gene organization among wheat and other plants (the unknown gene from wheat is not shown since no other plant had a similar gene). To find the location of the mTERF and PE genes, the relevant wheat DNA coding and derived amino acid sequences were used in BLAST searches to find the most similar sequences. If the best match was adjacent to the *LKR/SDH *gene, those matches are shown in Figure 6. The relative gene spacings are fairly conserved even with large differences in genome size, i.e., the PE, mTERF, and *LKR/SDH *genes share similar intergenic spacing despite as much as a ~30-fold difference in genome sizes, such as between *Arabidopsis *and one of the wheat genomes. Similar spacings occur in all other examined plant sequences which raises questions about the basis of genome size differences (gene-islands vs repetitive regions), and possible conserved functional clustering of genes. Figure 6 also shows that the conservation of the gene complement in this region with respect to the *LKR/SDH *gene, is not universal. All four monocot genomes contain the mTERF gene, but no dicot has an mTERF gene in this position of the genome. The PE gene is missing in *Brachypodium *but present in one copy in other available monocot sequences. Dicots show variation in the number of PE genes, with only one in this position in *Arabidopsis*, three each in *Medicago *and grape, and six in the current poplar assembly. Whether the difference in PE copy number is related to differential gene activity and function is unknown.

### Multiple transcripts from single LKR/SDH genes

Alternative transcript production from individual genes is a mechanism to expand potential protein diversity. This strategy can include both differential splicing of exons and multiple promoter sites, sometimes with the two in concert. The most extensive analyses have been with mammalian systems - where estimates are that more than half of the genes are involved in alternative splicing and nearly half have alternative promoters [36,37]. More limited analyses in plants indicate that upwards of 20% of plant genes are involved in alternative splicing [38,39]. An analysis of the conservation of alternative splicing between a dicot (*Arabidopsis*) and monocot (rice) concluded that since there was little conservation between the two plant groupings, this implied a limited role for alternative splicing in expanding the plant proteome [40]. However, even if specific alternative splicings are not conserved between dicots and monocots, this does not mean there are not important functional differences since such major differences in plant architecture, development, biochemistry, and genome organization are well-known.

The *LKR/SDH *gene, with its large number of exons, bifunctional nature, evidence of bi- and monofunctional transcripts, and diverse functional associations, would seem a good candidate for the study of multiple transcripts. The few reports on the relative abundance of monofunctional LKR or SDH mRNAs have not been consistent. It has been reported that the SDH mRNA is more abundant than LKR/SDH in *Arabidopsis *[10], a finding not evident in an earlier report [9]. In comparison, in mouse the LKR/SDH form was found more abundant than the SDH form [7]. The mouse study also failed to find evidence of a monofunctional LKR form. In plants, the only report of monofunctional LKR mRNAs is in cotton [9], although the authors speculate on the existence in other plants.

**Figure 7:**
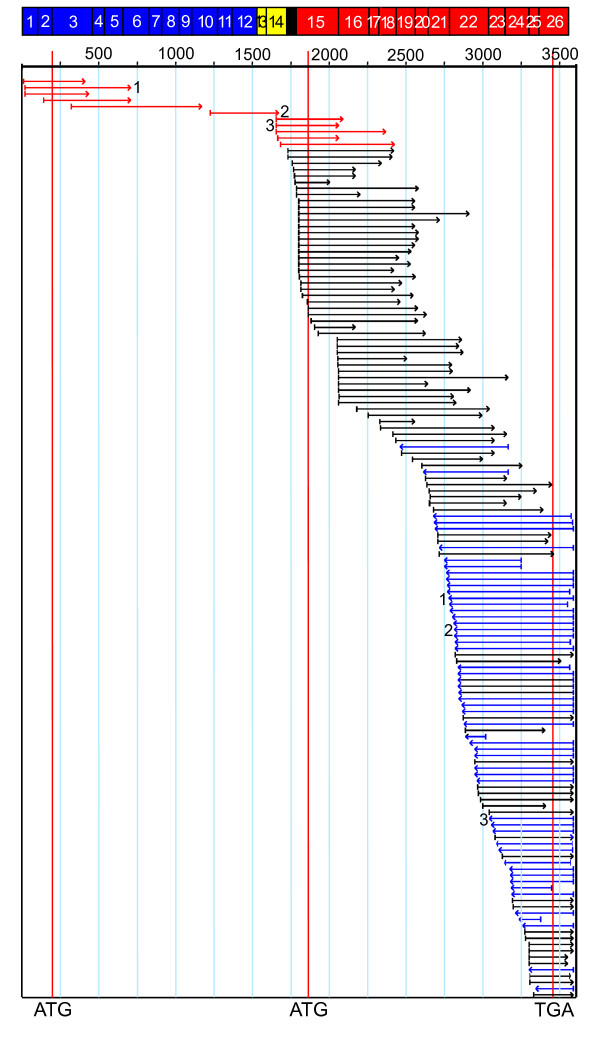
**Wheat LKR/SDH EST alignment**. The full-length LKR/SDH coding region derived from BAC 006M07 was used to align wheat ESTs matching with a BLASTn of e^-7 ^or lower. Red arrows indicate 5' reading ESTs initiating in the LKR or interdomain regions. For the remainder of the ESTs, black arrows show forward 5' reads and blue arrows show reverse 3' reads. Red vertical lines indicate the initial start codon of the LKR domain, the proposed start codon for a monofunctional SDH mRNA, and the common stop codon for both the bifunctional and SDH monofunctional mRNAs. Above the alignment are the numbered exons. Blue boxes indicate the LKR domain and red boxes the SDH domain. Yellow boxes are exons within the interdomain region. The black box represents the short sequence from intron 14 that is found at the 5' end of presumptive monofunctional SDH transcripts and not found in the red-arrowed ESTs. Three 5' and 3' EST pairs from the same clones are labeled as follows: 1, CJ882974 + CJ894783; 2, CJ881957 + CJ893808; 3, CJ883733 + CJ895693.

An analysis was carried out on three potential sources of multiple transcripts from the *LKR/SDH *gene - evidence for monofunctional LKR ESTs, monofunctional SDH ESTs, and alternative splicing using major collections of wheat and other plant ESTs. All available wheat LKR/SDH ESTs were aligned to the predicted full-length coding sequence (Figure 7; ESTs are shown as arrows and are in the same vertical order as the list of wheat LKR/SDH ESTs in Additional File 5). Of the 146 wheat ESTs, only 11 initiate in the LKR or linker domains (red arrows in Figure 7), while 135 ESTs match the SDH domain (black and blue arrows). Two observations suggest that most of the ESTs represent monofunctional SDH transcripts with only a few full-length bifunctional transcripts. If most of these SDH-domain ESTs were from bifunctional transcripts, there would be a gradation of 5' termini of the ESTs across the full-length sequence. Normally, ESTs from the 5' end of a sequence would be less represented for two reasons: longer transcripts, such as for LKR/SDH, will tend to be represented by more truncated clones during the cloning process, and since mRNAs are isolated via their 3' polyA sequences, 3' coding sequences in clones will be favored. Therefore, within a specific EST collection the ESTs will tend to terminate at the polyA site 3', and form a continuous pattern of truncated to full-length 5' termini. Figure 7 shows that instead of a gradual pattern, there is a relatively abrupt concentration of 5' ends around the area appropriate for the 5' end of monofunctional SDH transcripts. A second observation suggests a lack of wheat monofunctional LKR ESTs. All 11 ESTs that initiate within the LKR domain are 5' to 3' sequence reads with no EST reads consistent with 3' to 5' reads from the polyA end of mRNAs, as would be expected if there were LKR monofunctional ESTs in the existing wheat ESTs. Three of those 11 LKR ESTs also have 3' reads off the same clones (CJ882974 + CJ894783, CJ881951 + CJ893808, CJ883733 + CJ895693), and in all three cases the 3' sequence is from the 3' terminus of the SDH domain - thus confirming these three clones as originating from full-length LKR/SDH transcripts.

**Figure 8:**
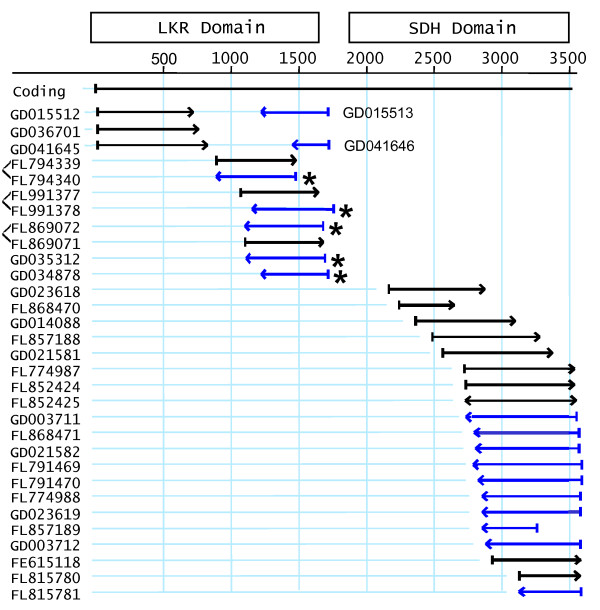
**Switchgrass EST alignment**. Switchgrass LKR/SDH ESTs are aligned to the maize coding sequence given in base pairs. Arrows indicate length of the ESTs and their direction of transcription. Black arrows are 5' reads and blue arrows are 3' reads. Regions of the LKR and SDH enzymatic domains are shown as boxes above the alignment. Paired 5' and 3' reads off the same cDNA clones are indicated either by ESTs on the same line or bracketed to the right of EST names. Asterisks indicated LKR ESTs with poly-A ends.

**Figure 9:**
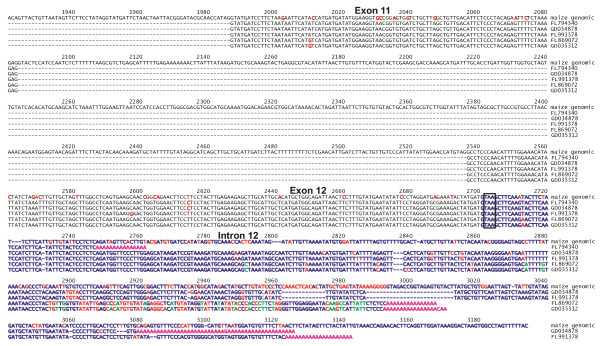
**Switchgrass LKR ESTs**. Five switchgrass ESTs that contain intron 12 sequence and a polyA tail are shown aligned to the maize LKR/SDH coding region. Sequences are given in black except for intron 12 which is in blue. The first stop codon for the reading frame into intron 12 is boxed. Differences among sequences are given in red and green for one or two differences to other sequences, respectively. PolyA tails of ESTs are shown in magenta.

Similar analyses were taken for ESTs from other plants (not shown). The sum result from rice, sorghum, maize, *Arabidopsis*, *Brachypodium*, *Medicago*, and barley are similar; i.e., a preponderance of SDH domain ESTs - 247 SDH and 64 LKR (of the 64 LKR, 36 are from rice and include a large number of ESTs of almost identical size from a restricted region of the LKR domain that may indicate some artifact in EST reporting). Of those plant LKR region ESTs, none are 3' reads - indicating bifunctional transcripts in these plants, but no evidence for LKR monofunctional transcripts. An exception was found in ESTs of switchgrass (*Panicum virgatum*) where there were 13 LKR ESTs and 20 SDH ESTs (Figure 8). Of the 13 LKR ESTs, there were five paired reads where clones were sequenced from both ends. One of those five pairs is from a chimeric cDNA clone since this 3' EST (GD015513) is from a hypothetical gene elsewhere in the genome. Three of these 3' ESTs from paired reads and two unpaired 3' read ESTs (asterisks in Figure 8) had poly-A sequences (Figure 9) - indicating support for monofunctional LKR mRNAs. All five 3' EST included sequences within intron 12 and contain a stop codon (boxed in Figure 9) near the position of the reported stop in the *Arabidopsis *(asterisk in Figure 2) monofunctional LKR sequence [9]. EST GD041646 also reads into intron 12, but is not shown since the sequence quality is poor. Thus, to generate switchgrass monofunctional LKR transcripts, instead of splicing out intron 12 at least part of the intron is retained and poly-A added. Whether the monofunctional LKR transcripts are the result of differential processing of a full-length LKR/SDH initial transcript or termination after transcribing the LKR domain is not confirmed, but we assume the latter. Note that the switchgrass LKR ESTs fall into two sequence classes with very similar sequences through the presumptive coding sequence, but diverging more 3' to the stop codon (Figure 9) - likely representing the two genomes of tetraploid switchgrass. No similar evidence was found for other plants, including *Brachypodium *which currently has the largest number of LKR/SDH ESTs (328) of any plant. None of those *Brachypodium *ESTs were in the LKR region. Similarly, BLAST analysis with the portion of the *Brachypodium *gene intron 12 resulted in no significant matches, and thus no evidence of *Brachypodium *LKR monofunctional sequences such as in switchgrass.

**Figure 10:**
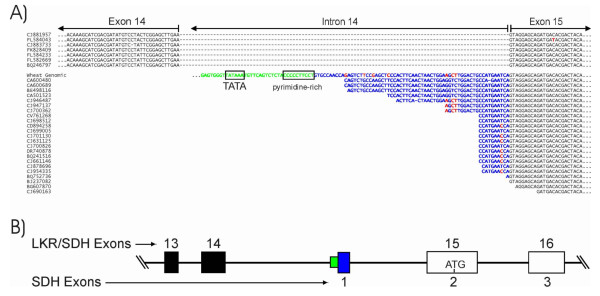
**Monofunctional wheat SDH ESTs**. (A) Wheat ESTs are aligned to the wheat consensus full-length LKR/SDH coding sequence. Intron 14 sequences are shown in blue and green. The sequences of the first seven ESTs have intron 14 spliced out (dashes indicate absent intron sequences). The rest of the ESTs begin with sequence from the middle of intron 14 (bases in blue) to form an exon that is spliced directly to exon 15 in those ESTs. Bases in green are the presumptive transcription initiation signal region. Base differences in red are assumed to indicate homoeologue LKR/SDH sequences of hexaploid wheat. (B) Diagram of exon/intron organization of LKR/SDH bifunctional and SDH monofunctional transcripts. Exons are shown by boxes. The SDH first exon sequence is within the LKR/SDH intron 14 and is shown by the blue box. The green box is the non-transcribed sequence indicated in frame A. Exon numbers are given above (LKR/SDH) and below (SDH) exon boxes.

The second monofunctional sequence report from the *LKR/SDH *gene is for the SDH domain only. As discussed previously, the wheat EST alignment in Figure 7 supports that most of the wheat LKR/SDH ESTs are from monofunctional SDH transcripts and a smaller number of bifunctional transcripts. Further support for these two transcript populations is given in Figure 10A where a segment of the wheat sequence alignment is shown to include two EST populations. The first seven EST sequences directly join exons 14 and 15, in agreement with consensus sequences of other plants. The remaining ESTs start with a sequence found within intron 14 (bases in blue). As shown in Figure 11B, the monofunctional SDH transcripts includes an exon not found in the full-length LKR/SDH transcript - an exon composed of sequence from the middle of intron 14 DNA of a full-length gene. This would be intron 1 of the monofunctional SDH sequence and separated from the following exon (exon 15 of the complete gene) by 524 bp with splice canonical junctions (GT...AG). Consistent with polymerase II initiation sites [41], a presumptive TATA box (TATAA) is at -34 bp to the 5' end of the wheat ESTs with the most 5' matching sequences (Figure 10A). Between the TATA box and the EST sequences is a pyrimidine-rich segment - again consistent with transcription initiation sites [41]. Once the SDH transcript is spliced, the first ATG codon that allows a reading frame consistent with an SDH sequence occurs in the middle of SDH exon 2 (LKR/SDH exon 15) as indicated in Figure 10B and the downward arrowhead at position 587 of Figure 2. This position is consistent with the translation initiation site reported for *Arabidopsis *(upward arrow at position 588 in Figure 2), but is in contrast to a previous report [12] that predicted the maize monofunctional SDH transcript used a TATA box at the end of exon 15 and initiated translation near the beginning of exon 16. However, there was no EST support of those previously reported predictions.

Besides wheat, only two other plant species' EST collection contained sequences consistent with transcript initiation within intron 14. One of those was *Brachypodium *as shown in Additional File 11A. Four *Brachypodium *ESTs (CCXG11317, CCXG13127, CCXO11098, CCXG8102) have identical 5' ends that begin immediately following the pyrimidine-rich region and may represent the actual start site for *Brachypodium *monofunctional SDH transcripts. Those four ESTs plus six other *Brachypodium *ESTs all match the junction of the intron 14-derived monofunctional SDH first exon to SDH exon 2 (LKR/SDH exon 15) as show in Additional File 11B - and match the same structure as with wheat (Figure 10). Finally, although there are only a few Poplar LKR/SDH ESTs, they also support the existence of both bifunctional and SDH monofunctional transcripts. ESTs DV465683 and DY800647 have 5' sequences reading from intron 12 sequence into exon 13, suggesting SDH monofunctional transcript. Poplar ESTs CX180963 and CN520125 read directly from exon 12 into exon 13 with no intervening intron 12 sequence, supporting a bifunctional LKR/SDH transcript (not shown).

The lack of EST support for monofunctional mRNAs in many systems does not mean they do not exist, but only that the EST resources do not support them. However, it does continue to support a preponderance of SDH transcripts, bi- and monofunctional, which suggests differential contributions of the LKR and SDH domains to plant cell metabolism.

Finally, the analysis of plant ESTs failed to convincing support for major multiple populations of alternatively spliced transcripts for the 25 dicot and 26 monocot *LKR/SDH *exons (exceptions being the monofunctional transcripts described above). Small numbers of differential splicing were found in several plants (not shown), but none in sufficient numbers to suggest differential roles in plant cell metabolism rather than examples of aberrant splicings with no functional roles. For example, a close examination of the wheat ESTs suggests a small number of such alternative splicings. The five wheat ESTs that cover the region that includes the bifunctional consensus start codon represent three sequences - presumably from the three hexaploid wheat genomes. ESTs BJ266925 and CJ702289 match the BAC A-genome sequence exactly, while CJ882974 is a second sequence and FL577869 plus BJ248520 represents the third sequence. Although EST BJ266925 spans the start codon region of the BAC sequences, it, unlike the other four ESTs, does not encode the same ATG codon - exon 2 is missing, with the splice going from the end of exon 1 to the beginning of exon 3. In another wheat example, three pairs of ESTs (reads from both ends) show multiple variant splicing at the 3' end of the SDH sequence: CJ965444+CJ953360; CJ950703+CJ962606; CJ567209+CJ6741282. These three different original cDNAs continue transcription into post-exon-26 genomic sequence, and differentially splice previous sequence after exon 23 (not shown). No obvious consensus splice site sequences are evident, but the three sequences use at least one different splice site from other cDNAs. More in-depth EST sequencing of more plants should clarify the existence and possible roles of specific alternative splicings.

## Conclusion

The isolation and characterization of a segment of the wheat genome containing the *LKR/SDH *gene is shown. The wheat LKR/SDH genomic segment was found to originate from the A-genome of wheat, and EST analysis indicates all three *LKR/SDH *genes in hexaploid wheat are transcriptionally active, at least for monofunctional SDH transcripts. Comparative analyses with other plant *LKR/SDH *genes and ESTs shows conservation of the basic exon/intron organization between the wheat gene and previously analyzed genes from maize and *Arabidopsis *and previously unanalyzed genes from rice, *Medicago*, grape, poplar, sorghum, and *Brachypodium*. Relative conservation of exon+intron length, even in plants whose genome sizes differ by 30-fold or more, further supports the intergenic regions as sites of genome expansion. Exceptions to the general gene length conservation are *Arabidopsis *and grape, whose *LKR/SDH *genes are shorter and longer, respectively, due to shorter and longer intron lengths. For *Arabidopsis*, the smaller introns are consistent with the general compactness of the *Arabidopsis *genome. However, the basis and functionality of larger grape introns is not consistent with genes from plants with similarly-sized genomes. Both the structure of the *LKR/SDH *gene and the sets of immediately adjacent genes within the genome show lineage-specific differences between monocots and dicots, including different gene positionings and different copy numbers of an adjacent pectinesterase gene. Two findings suggest variation in structure and activity of *LKR/SDH *genes among plants. First, although most plants seem to contain a single conserved *LKR/SDH *gene, poplar possesses multiple genes. Second, there are differences among plants in evidence for bifunctional and monofunctional LKR and SDH transcripts among the available EST data. The analyses of ESTs provides some of the most detailed data for multiple transcripts from a single gene, particularly evidence for monofunctional LKR transcripts in switchgrass and monofunctional SDH transcripts in wheat and *Brachypodium*. There is also evidence in these plants that the monofunctional LKR transcripts read into an intron of the full-length sequence, and for an additional exon for SDH transcripts composed of a central portion of a full-length intron. The lack of similar EST evidence in other species may be due to sampling differences in EST production, but also may indicate fundamental differences in LKR/SDH control and function.

## Methods

### BAC isolation and sequencing

A BAC library of wheat tetraploid *T. turgidum *ssp. *durum *(2n - 4× = 28, AABB) cultivar Langdon [42] was screened using a mixed probe composed of two wheat EST clones encoding portions of the SDH domain (BE428366 and BE498116) and a maize full-length LKR/SDH cDNA clone (NM_001111403) obtained from P. Arruda [12]. Twelve BACs were isolated and further characterized by Southern analysis and BAC fingerprinting to represent two distinct sequences. BAC 0006M07 was selected for sequencing based on its central position in one contig and apparent central location of the LKR/SDH sequence and was sized at about 160,000 bp. Sequencing of BAC 0006M07 was carried out to a depth of about 20× by procedures described in detail elsewhere [27]. Briefly, randomly shear BAC DNA was blunt-ended with mung bean exonuclease (BioLab), dephosphyorylated with shrimp alkaline phosphatase (USB), single A-tailed with *Taq *polymerase, and the resulting DNA fractionated to 3-5 kb with agarose gels and the Qiagen Gel Extraction Kit. This DNA was used to generate shotgun libraries using the vector pCR4TOPO and transformed into DH10B electroMAX cells (Invitrogen). Randomly picked clones were sequenced at both insert ends with T3 and T7 primers and BigDye chemistry (Applied Biosystems) with an ABI3730×l sequencer.

Sequence analysis began with contig assembly using both Phrap http://www.phrap.org and the Lasergene SeqMan module http://www.DNAStar.com. Gaps and uncertain sequences were resolved by comparing the assemblies from the two software packages and primer walking. Regions of less coverage or ambiguous reads were rechecked with primers designed to cover those regions.

### Analysis of sequences

NCBI http://www.ncbi.nlm.nih.gov was used for annotation of the new wheat BAC sequence by BLAST analyses and total EST analyses by direct querying to NCBI. Exon/intron junctions are predicted by alignment with Triticeae EST sequences, when available, or with other monocot EST if no Triticeae ESTs covered those sequences.

Sources of genomic sequences were as follows: *Arabidopsis thaliana LKR/SDH*, Genbank ATU95759; *Brachypodium distachyon*, http://brachypodium.org; *Medicago truncatula*, http://www.tigr.org/tdb/e2k1/mta1/; poplar (*Populus trichocarpa*), http://genome.jgi-psf.org; sorghum (*Sorghum bicolor*), http://genome.jgi-psf.org; grape (*Vitis vinifera*), http://www.genoscope.cns.fr/externe/GenomeBrowser/Vitis/; rice (*Oryza sativa*), http://gramene.org, MSU-TIGR pseudomolecule assembly release 5 of IRGSP (The International Rice Genome Sequencing Project) and Genbank AP004849. BAC sequences from Genbank were as follows: cotton (*Glossypium hirsutum*), AF264146; maize (*Zea Mays*), AF271636; poplar, AC209229. The *Brachypodium *sequence data were produced by the US Department of Energy Joint Genome Institute http://www.jgi.doe.gov/. For ease of reading, it will be understand that common names and genus names will be used unless referring to different species than noted above; e.g., *Brachypodium *instead of *B. distachyon *and rice instead of *O. sativa*. Plant ESTs were searched at Genbank, except for *Brachypodium *ESTs that were found at brachypodium.org. Determination of coding sequences and exon/intron junctions were accomplished by comparing genomic DNAs to ESTs and cDNA clones from the same plant, or where necessary, comparing to ESTs and cDNAs from closely related plants.

### PCR primers for genome identification

The sequence of the rice LKR/SDH region from BAC AP004849 was compared to the wheat BAC 0006M07. Primer pairs were designed from conserved regions and tested against genomic DNA of a series of diploid, tetraploid, and hexaploid wheats and wheat ancestors. Primer pair F3 (AAAGAAGCATCTACCGTATATAGG) and R3 (TTCATGGTGGAGCAGTACCATATC) amplified the expected fragment size in all wheat DNAs including DNA from the A, AB, D, and ABD genomes. PCR products were sequenced from all these genomes and the sequences compared. Unique bases were used to design single genome-specific primers for the A, B, and D genomes: A genome, primer AF3 GCATTCAGTGTTATTTGCCAATGT; B genome, primer BF3 CTCCACATCTAACACAAAGATATAC; D genome, primer DF3 GGATTTTTCTCAATGACCTCCTTG.

### Phylogenetic analysis of LKR/SDH proteins

A phylogenetic analysis of LKR/SDH proteins was carried out using the MEGA4 software package [43]. A protein alignment used ClustalW and the evolutionary relationship inferred by the Neighbor-Joining method [44]. A bootstrap test was used to determine the percentage of replicate trees in which the associated taxa clustered together [45]. Evolutionary distances were computed using the Poisson correction method [46] and are in the units of the number of amino acid substitutions per site.

## Authors' contributions

ODA was lead author in planning, analysis, and manuscript preparation. DCD carried out the BAC annotation and PCR experiments. SH screened the BAC library. DCD and SH carried out the sequencing. YG contributed to planning and analysis. All authors contributed, read, and approved the manuscript.

## Supplementary Material

Additional File 1 **Pair-wise distances of LKR/SDH proteins**. Evolutionary relationship of full-length plant LKR/SDH coding regions. Click here for file

Additional File 2 **Intron conservation and divergence**. The wheat LKR/SDH introns were compared to the *Brachypodium *and maize genes from start to stop. Click here for file

Additional File 3 **Bin-mapping the wheat *LKR/SDH *gene**. EST BE606591 was bin-mapped to the long arm of wheat chromosomes 6A and 6B. Click here for file

Additional File 4 **Genome origin of BAC 0006M07**. DNA fragments were amplified from DNA the wheat BAC and several wheat genetic germplasms. Click here for file

Additional File 5 **Wheat LKR/SDH ESTs**. The list of currently publicly available wheat LKR/SDH ESTs. Click here for file

Additional File 6 **Wheat LKR/SDH EST contigs**. Wheat ESTs containing LKR/SDH sequences were assembled and compared to the BAC LKR/SDH coding and 3'-UTR sequence. Click here for file

Additional File 7 **Wheat ESTs aligning to BAC 0006M07 pectinesterase gene**. Wheat pectinesterase ESTs are aligned to the wheat BAC. Click here for file

Additional File 8 **Amino acid alignment of mTERF proteins**. The wheat BAC mTERF protein is aligned to the best matches from rice and maize. Click here for file

Additional File 9 **Unknown gene aligned with wheat ESTs**. The unknown wheat gene region matching barley ESTs is aligned with those ESTs. Click here for file

Additional File 10 **ESTs match two distinct poplar 3' UTRs**. Poplar ESTs aligned to the 3' UTRs of poplar *LKR/SDH *genes 1 and 2. Click here for file

Additional File 11 **Monofunctional *Brachypodium *SDH ESTs**. Brachypodium ESTs are aligned to Brachypodium and wheat LKR/SDH exon and intron sequences. Click here for file
