# Supplementary material for: Structural and transcriptional analysis of plant genes encoding the bifunctional lysine ketoglutarate reductase saccharopine dehydrogenase enzyme
Source: BMC Plant Biol. 2010 Jun 16;10:113. doi: 10.1186/1471-2229-10-113 (PMC3017810; doi:10.1186/1471-2229-10-113)
Supplement: Additional File 1 — Pair-wise distances of LKR/SDH proteins. Evolutionary relationship of full-length plant LKR/SDH coding regions. [file 1471-2229-10-113-S1.DOC]

**Additional File 1 Pair-wise distances of LKR/SDH full-length proteins.**

Amino acid sequences were aligned with ClustalW and the evolutionary relationship inferred by the Neighbor-Joining method. Evolutionary distances were computed using the Poisson correction method and are in the units of the number of amino acid substitutions per site.

No. of Taxa : 11

Data File : C:\Program Files\MEGA 4\Examples\LKR 19Feb10MEGAformatc.meg

Data Title : testc

Data Type : Amino acid

Analysis : Pairwise distance calculation

->Compute : Distances only

Include Sites : ==============================

->Gaps/Missing Data : Complete Deletion

Substitution Model : ==============================

->Model : Amino: Poisson correction

->Substitutions to Include : All

->Pattern among Lineages : Same (Homogeneous)

->Rates among sites : Uniform rates

No. of Sites : 984

d : Estimate

[ 1] wheat

[ 2] Poplar-1

[ 3] Poplar-2

[ 4] maize

[ 5] cotton

[ 6] Arabidopsis

[ 7] Brachypodium

[ 8] moss

[ 9] Medicago

[10] rice

[11] grape

[ 1 2 3 4 5 6 7 8 9 10 11 ]

[ 1]

[ 2] 0.367

[ 3] 0.364 0.018

[ 4] 0.168 0.374 0.372

[ 5] 0.362 0.264 0.269 0.371

[ 6] 0.375 0.333 0.336 0.383 0.321

[ 7] 0.074 0.368 0.365 0.170 0.364 0.375

[ 8] 0.591 0.630 0.619 0.591 0.628 0.638 0.593

[ 9] 0.374 0.284 0.285 0.375 0.281 0.336 0.378 0.634

[10] 0.132 0.365 0.359 0.161 0.364 0.381 0.131 0.607 0.370

[11] 0.359 0.236 0.239 0.353 0.257 0.326 0.365 0.607 0.261 0.349
