# Supplementary material for: Structural and transcriptional analysis of plant genes encoding the bifunctional lysine ketoglutarate reductase saccharopine dehydrogenase enzyme
Source: BMC Plant Biol. 2010 Jun 16;10:113. doi: 10.1186/1471-2229-10-113 (PMC3017810; doi:10.1186/1471-2229-10-113)
Supplement: Additional File 2 — Intron conservation and divergence. The wheat LKR/SDH introns were compared to the Brachypodium and maize genes from start to stop. [file 1471-2229-10-113-S2.PPT]

## Slide 1
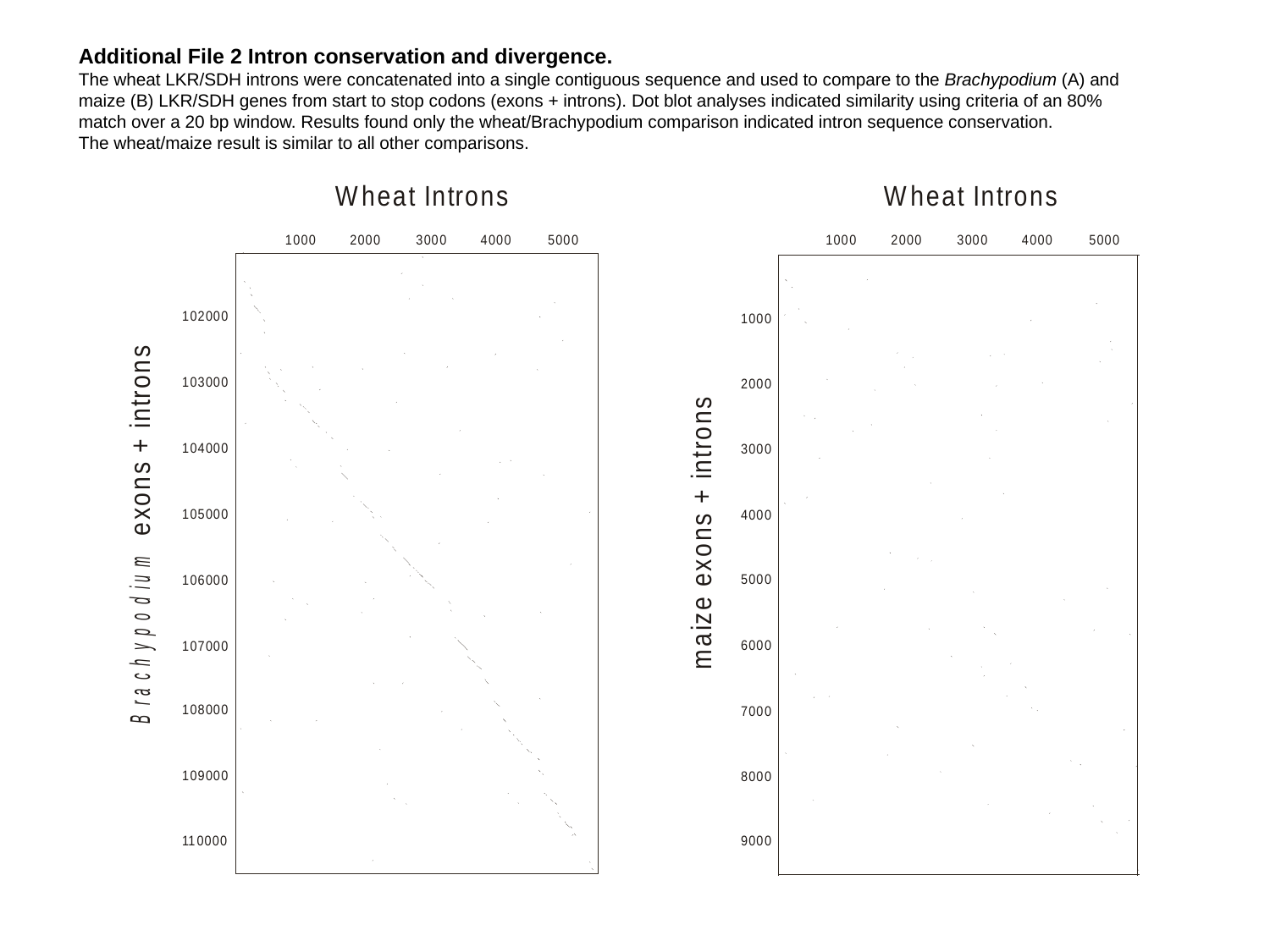

Additional File 2 Intron conservation and divergence.
The wheat LKR/SDH introns were concatenated into a single contiguous sequence and used to compare to the Brachypodium (A) and
maize (B) LKR/SDH genes from start to stop codons (exons + introns). Dot blot analyses indicated similarity using criteria of an 80%
match over a 20 bp window. Results found only the wheat/Brachypodium comparison indicated intron sequence conservation.
The wheat/maize result is similar to all other comparisons.
