# Supplementary material for: Structural and transcriptional analysis of plant genes encoding the bifunctional lysine ketoglutarate reductase saccharopine dehydrogenase enzyme
Source: BMC Plant Biol. 2010 Jun 16;10:113. doi: 10.1186/1471-2229-10-113 (PMC3017810; doi:10.1186/1471-2229-10-113)
Supplement: Additional File 3 — Bin-mapping the wheat LKR/SDH gene. EST BE606591 was bin-mapped to the long arm of wheat chromosomes 6A and 6B. [file 1471-2229-10-113-S3.PPT]

## Slide 1
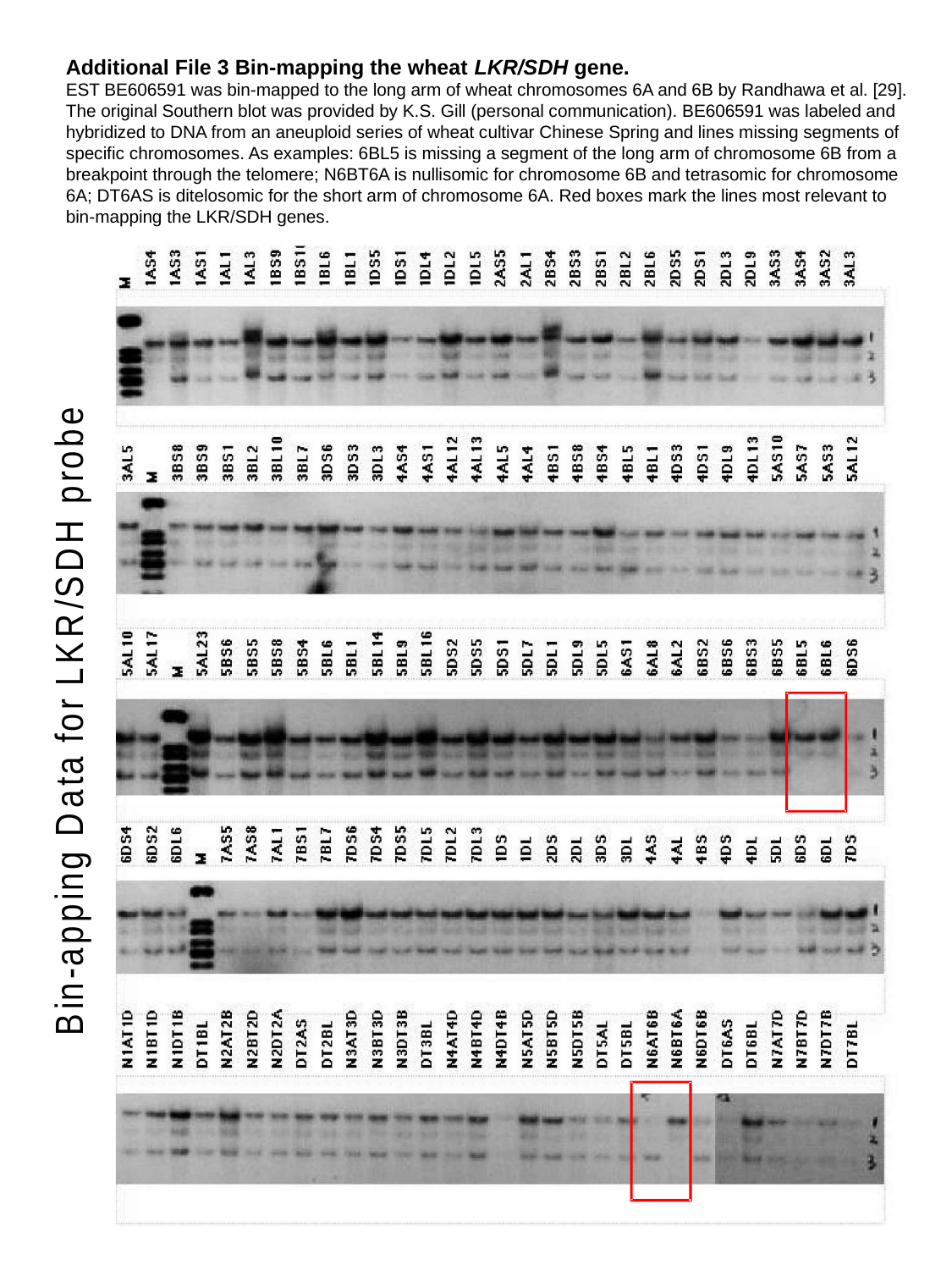

Additional File 3 Bin-mapping the wheat LKR/SDH gene.
EST BE606591 was bin-mapped to the long arm of wheat chromosomes 6A and 6B by Randhawa et al. [29].
The original Southern blot was provided by K.S. Gill (personal communication). BE606591 was labeled and
hybridized to DNA from an aneuploid series of wheat cultivar Chinese Spring and lines missing segments of
specific chromosomes. As examples: 6BL5 is missing a segment of the long arm of chromosome 6B from a
breakpoint through the telomere; N6BT6A is nullisomic for chromosome 6B and tetrasomic for chromosome
6A; DT6AS is ditelosomic for the short arm of chromosome 6A. Red boxes mark the lines most relevant to
bin-mapping the LKR/SDH genes.
