# Supplementary material for: Structural and transcriptional analysis of plant genes encoding the bifunctional lysine ketoglutarate reductase saccharopine dehydrogenase enzyme
Source: BMC Plant Biol. 2010 Jun 16;10:113. doi: 10.1186/1471-2229-10-113 (PMC3017810; doi:10.1186/1471-2229-10-113)
Supplement: Additional File 4 — Genome origin of BAC 0006M07. DNA fragments were amplified from DNA the wheat BAC and several wheat genetic germplasms. [file 1471-2229-10-113-S4.PPT]

## Slide 1
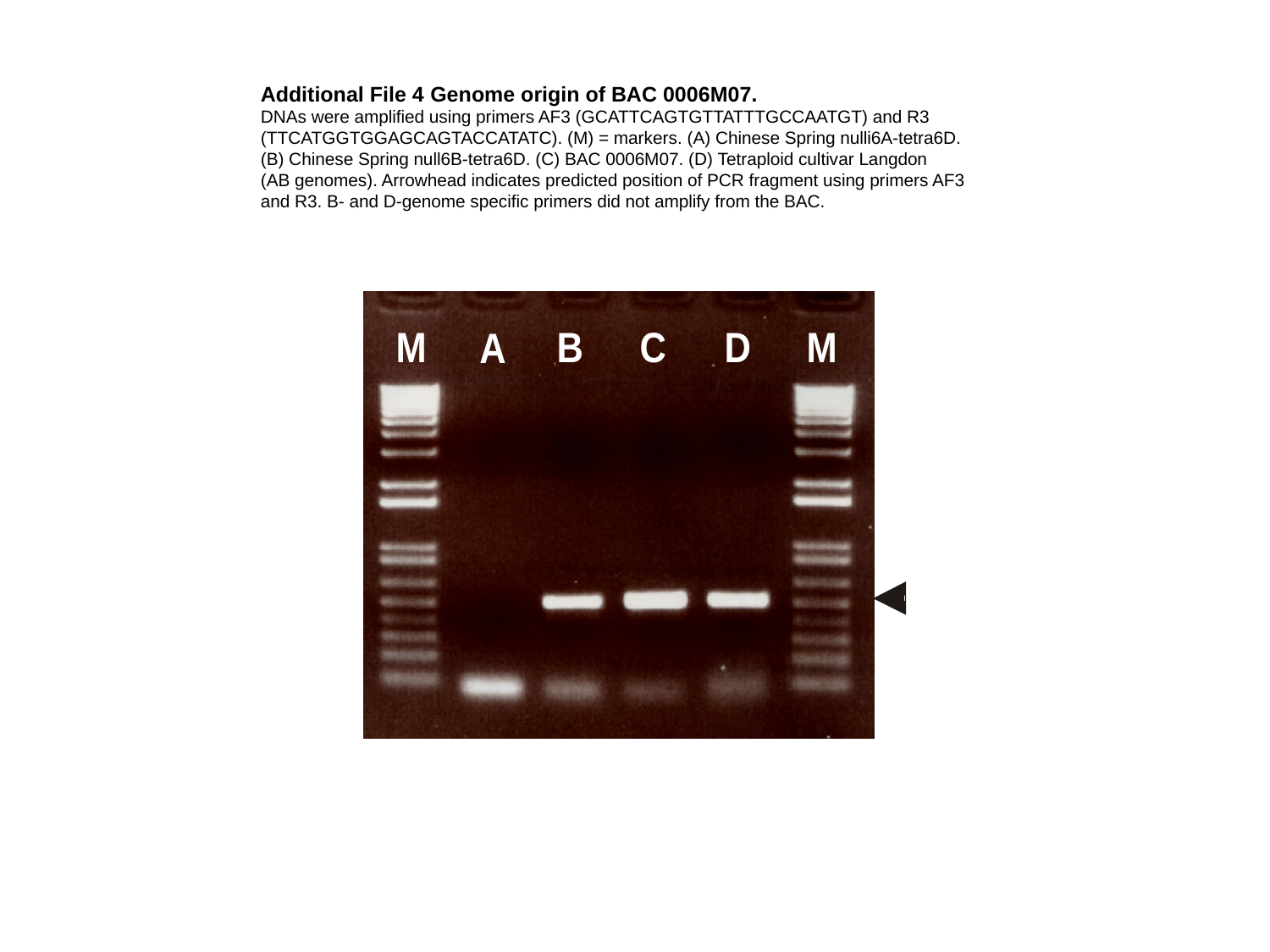

Additional File 4 Genome origin of BAC 0006M07.
DNAs were amplified using primers AF3 (GCATTCAGTGTTATTTGCCAATGT) and R3
(TTCATGGTGGAGCAGTACCATATC). (M) = markers. (A) Chinese Spring nulli6A-tetra6D.
(B) Chinese Spring null6B-tetra6D. (C) BAC 0006M07. (D) Tetraploid cultivar Langdon
(AB genomes). Arrowhead indicates predicted position of PCR fragment using primers AF3
and R3. B- and D-genome specific primers did not amplify from the BAC.
