# Supplementary material for: Structural and transcriptional analysis of plant genes encoding the bifunctional lysine ketoglutarate reductase saccharopine dehydrogenase enzyme
Source: BMC Plant Biol. 2010 Jun 16;10:113. doi: 10.1186/1471-2229-10-113 (PMC3017810; doi:10.1186/1471-2229-10-113)
Supplement: Additional File 5 — Wheat LKR/SDH ESTs. The list of currently publicly available wheat LKR/SDH ESTs. [file 1471-2229-10-113-S5.DOC]

## Additional File 5 Wheat LKR/SDH ESTs.

The list of currently publicly available wheat LKR/SDH ESTs. Criterion for identification was a tBLASTn expectation value of e^-7^ or less. Three ESTs were too short to assign to genomes and were not included.

FK826981

CJ882974

BJ248520

CJ702289

CA693672

CJ881957

FL584043

CJ883733

FK828409

FL584233

FL582669

BQ246797

CA600480

CA600689

BE498116

CA501523

CJ946487

CJ947137

CJ700362

CV761268

CJ698512

CD894258

CJ699005

CJ701130

CJ631125

CJ700826

DR740878

BQ241516

CJ661146

CJ878696

CJ954335

BQ752736

BJ237082

BG607870

CJ690163

CJ701941

CJ873113

CJ873409

CA498211

BQ245460

CJ702395

FD468624

FD468629

FD468317

FD468639

FD468497

FD468421

FD468282

FD468347

FK828462

FL582632

FD468281

CJ659779

CJ875837

CV770546

CJ698109

BQ483922

FL584197

BG906544

FM208478

BF200255

BQ905440

CA595081

CJ727980

CJ634853

BJ260450

CJ874752

CD925974

CJ884658

CJ553618

CJ594947

CJ904851

CJ904577

CJ959174

CV776265

FL578455

FK826942

CJ624195

CJ552189

CJ522549

CJ592104

CJ887772

CJ886284

CJ894783

CJ596497

CJ596613

CJ890629

CJ916356

CJ893808

CJ916627

CD921622

CJ880817

CJ592532

CJ958307

CJ583736

CJ884922

CJ892806

CJ966373

CJ549623

CJ593060

CJ657334

BJ242625

CJ809222

CJ891722

CJ526360

BE606591

CD492116

CJ885226

CJ594469

BJ266147

CJ592226

CJ698226

CJ698227

AM285515

BE428366

CD883409

CJ895693

CD453906

CJ596093

CJ701913

CJ953360

BJ272037

BQ802995

CJ950703

CJ967416

CO348941

CA664230

CJ596128

BQ238437

CJ955515

CA688800

BG906545

BJ266316

CJ968174

CJ956001

FD468410

FD469012

FD468790

FL578917

FK827315

EB514198

BQ801258

AL825962

CJ962693

CJ950502
