# Supplementary material for: Structural and transcriptional analysis of plant genes encoding the bifunctional lysine ketoglutarate reductase saccharopine dehydrogenase enzyme
Source: BMC Plant Biol. 2010 Jun 16;10:113. doi: 10.1186/1471-2229-10-113 (PMC3017810; doi:10.1186/1471-2229-10-113)
Supplement: Additional File 6 — Wheat LKR/SDH EST contigs. Wheat ESTs containing LKR/SDH sequences were assembled and compared to the BAC LKR/SDH coding and 3'-UTR sequence. [file 1471-2229-10-113-S6.PPT]

## Slide 1
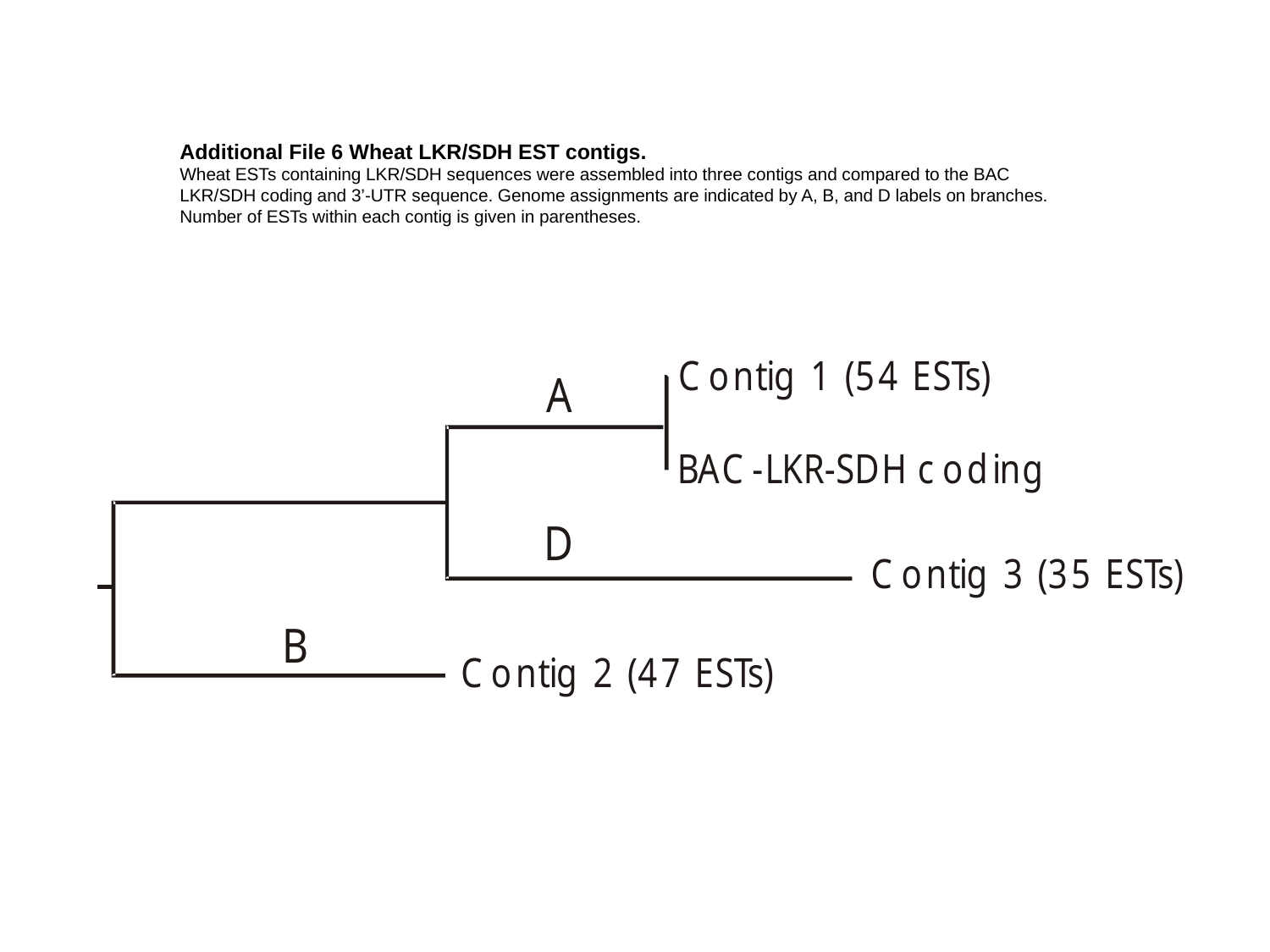

Additional File 6 Wheat LKR/SDH EST contigs.
Wheat ESTs containing LKR/SDH sequences were assembled into three contigs and compared to the BAC
LKR/SDH coding and 3’-UTR sequence. Genome assignments are indicated by A, B, and D labels on branches.
Number of ESTs within each contig is given in parentheses.
