# Supplementary material for: Structural and transcriptional analysis of plant genes encoding the bifunctional lysine ketoglutarate reductase saccharopine dehydrogenase enzyme
Source: BMC Plant Biol. 2010 Jun 16;10:113. doi: 10.1186/1471-2229-10-113 (PMC3017810; doi:10.1186/1471-2229-10-113)
Supplement: Additional File 7 — Wheat ESTs aligning to BAC 0006M07 pectinesterase gene. Wheat pectinesterase ESTs are aligned to the wheat BAC. [file 1471-2229-10-113-S7.PPT]

## Slide 1
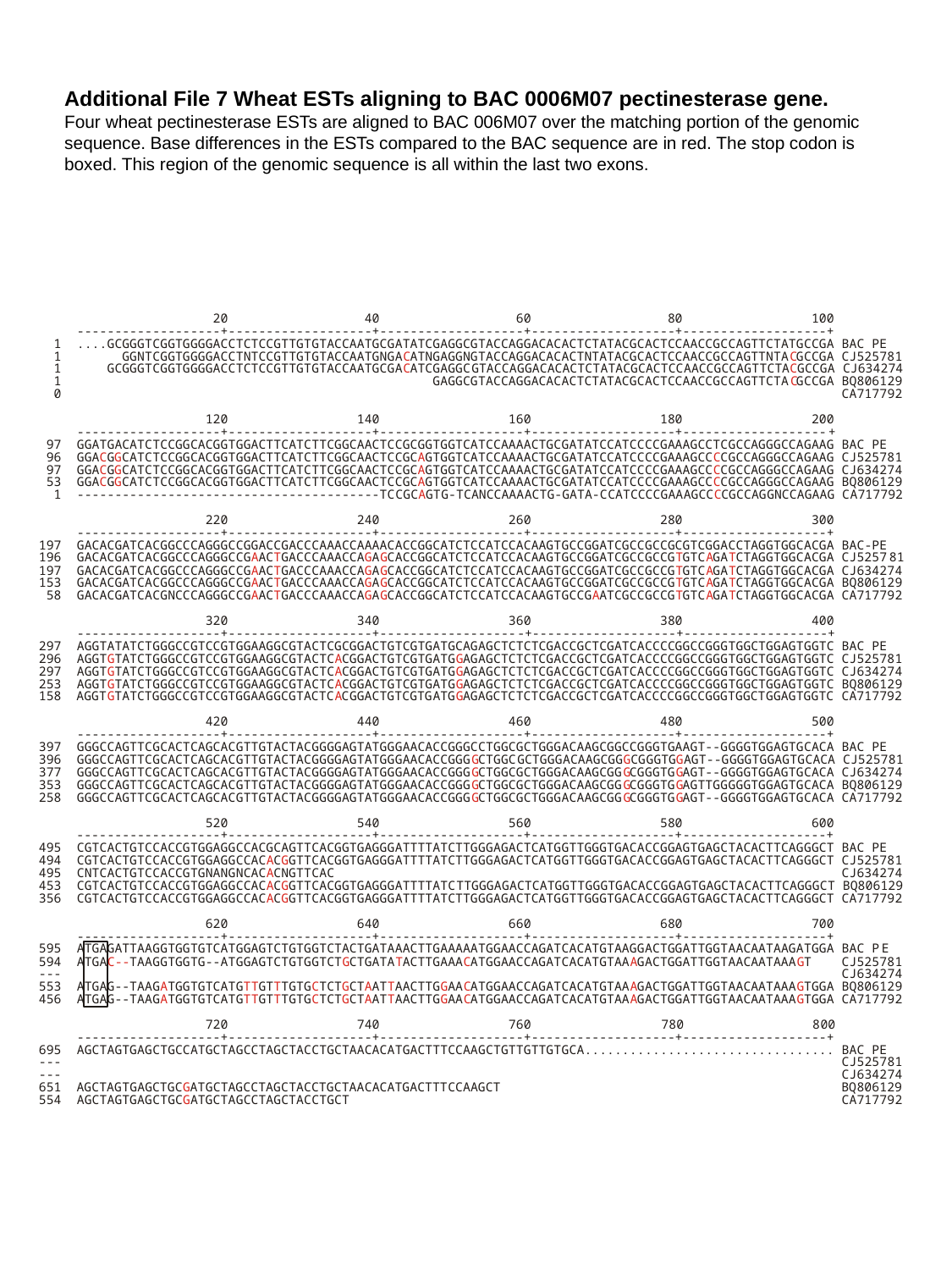

Additional File 7 Wheat ESTs aligning to BAC 0006M07 pectinesterase gene.
Four wheat pectinesterase ESTs are aligned to BAC 006M07 over the matching portion of the genomic
sequence. Base differences in the ESTs compared to the BAC sequence are in red. The stop codon is
boxed. This region of the genomic sequence is all within the last two exons.
