# Supplementary material for: Structural and transcriptional analysis of plant genes encoding the bifunctional lysine ketoglutarate reductase saccharopine dehydrogenase enzyme
Source: BMC Plant Biol. 2010 Jun 16;10:113. doi: 10.1186/1471-2229-10-113 (PMC3017810; doi:10.1186/1471-2229-10-113)
Supplement: Additional File 8 — Amino acid alignment of mTERF proteins. The wheat BAC mTERF protein is aligned to the best matches from rice and maize. [file 1471-2229-10-113-S8.PPT]

## Slide 1
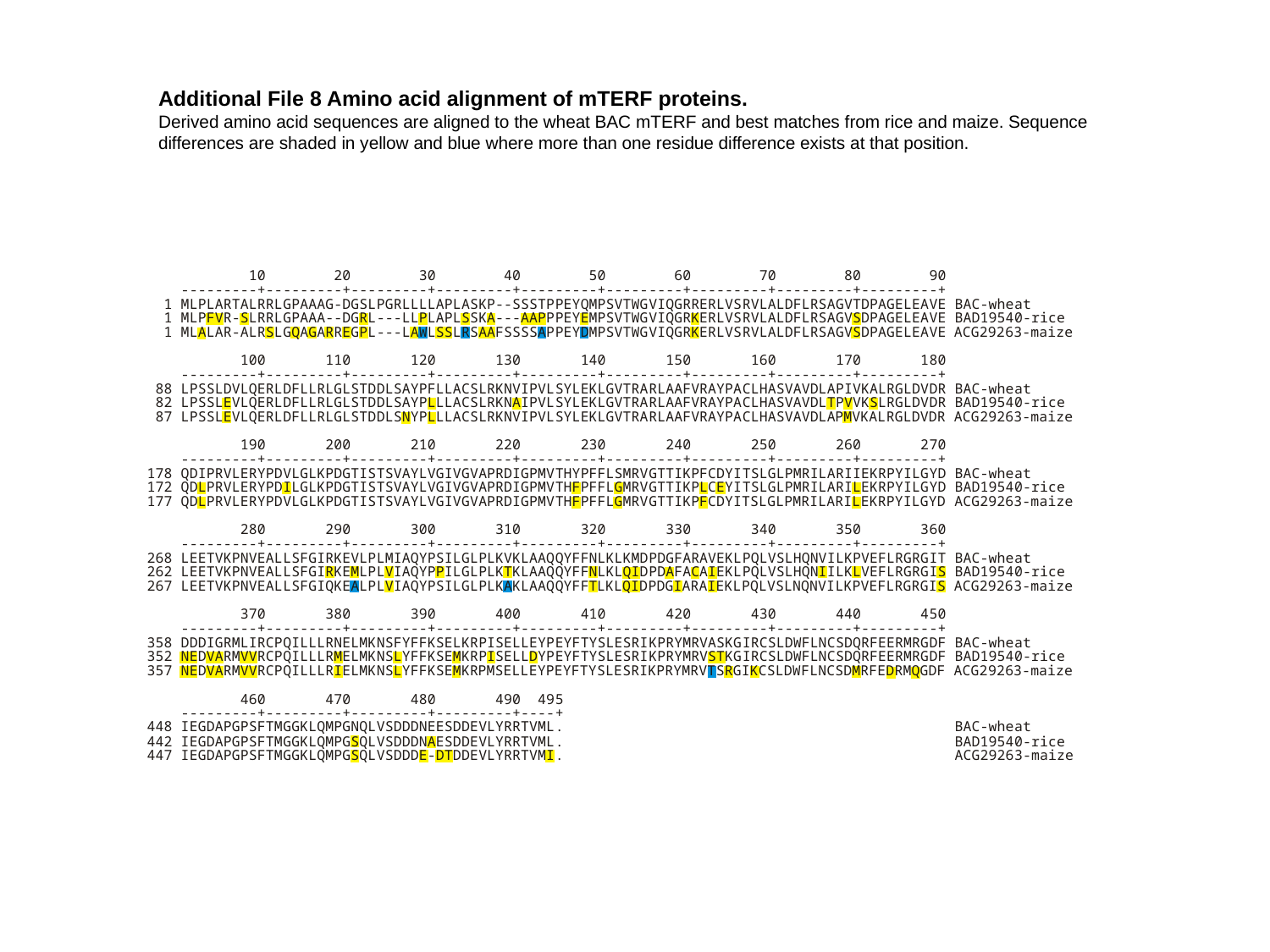

Additional File 8 Amino acid alignment of mTERF proteins.
Derived amino acid sequences are aligned to the wheat BAC mTERF and best matches from rice and maize. Sequence
differences are shaded in yellow and blue where more than one residue difference exists at that position.
