# Supplementary material for: Structural and transcriptional analysis of plant genes encoding the bifunctional lysine ketoglutarate reductase saccharopine dehydrogenase enzyme
Source: BMC Plant Biol. 2010 Jun 16;10:113. doi: 10.1186/1471-2229-10-113 (PMC3017810; doi:10.1186/1471-2229-10-113)
Supplement: Additional File 9 — Unknown gene aligned with wheat ESTs. The unknown wheat gene region matching barley ESTs is aligned with those ESTs. [file 1471-2229-10-113-S9.PPT]

## Slide 1
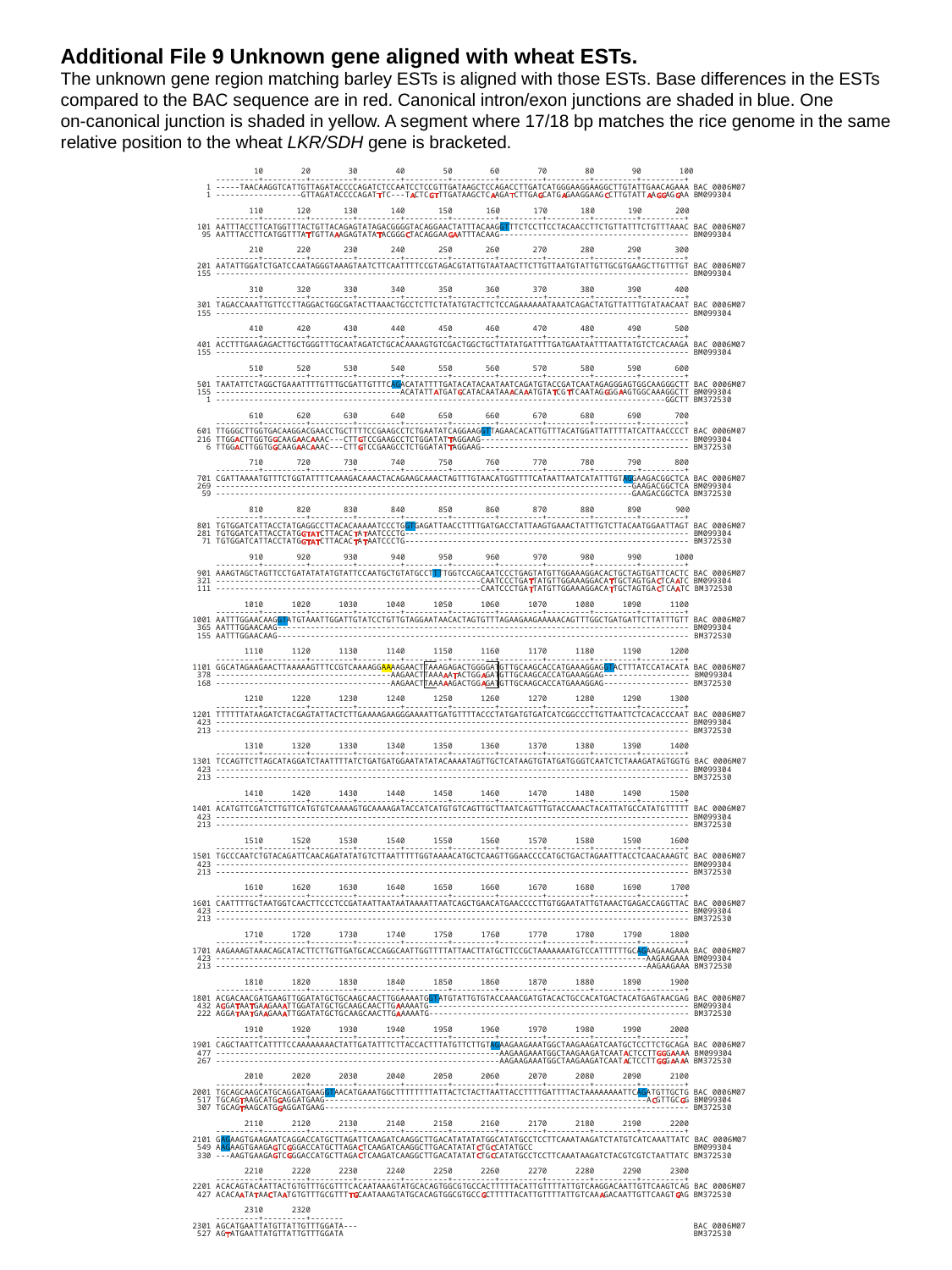

Additional File 9 Unknown gene aligned with wheat ESTs.
The unknown gene region matching barley ESTs is aligned with those ESTs. Base differences in the ESTs
compared to the BAC sequence are in red. Canonical intron/exon junctions are shaded in blue. One
on-canonical junction is shaded in yellow. A segment where 17/18 bp matches the rice genome in the same
relative position to the wheat LKR/SDH gene is bracketed.
