# Supplementary material for: Structural and transcriptional analysis of plant genes encoding the bifunctional lysine ketoglutarate reductase saccharopine dehydrogenase enzyme
Source: BMC Plant Biol. 2010 Jun 16;10:113. doi: 10.1186/1471-2229-10-113 (PMC3017810; doi:10.1186/1471-2229-10-113)
Supplement: Additional File 10 — ESTs match two distinct poplar 3' UTRs. Poplar ESTs aligned to the 3' UTRs of poplar LKR/SDH genes 1 and 2. [file 1471-2229-10-113-S10.PPT]

## Slide 1
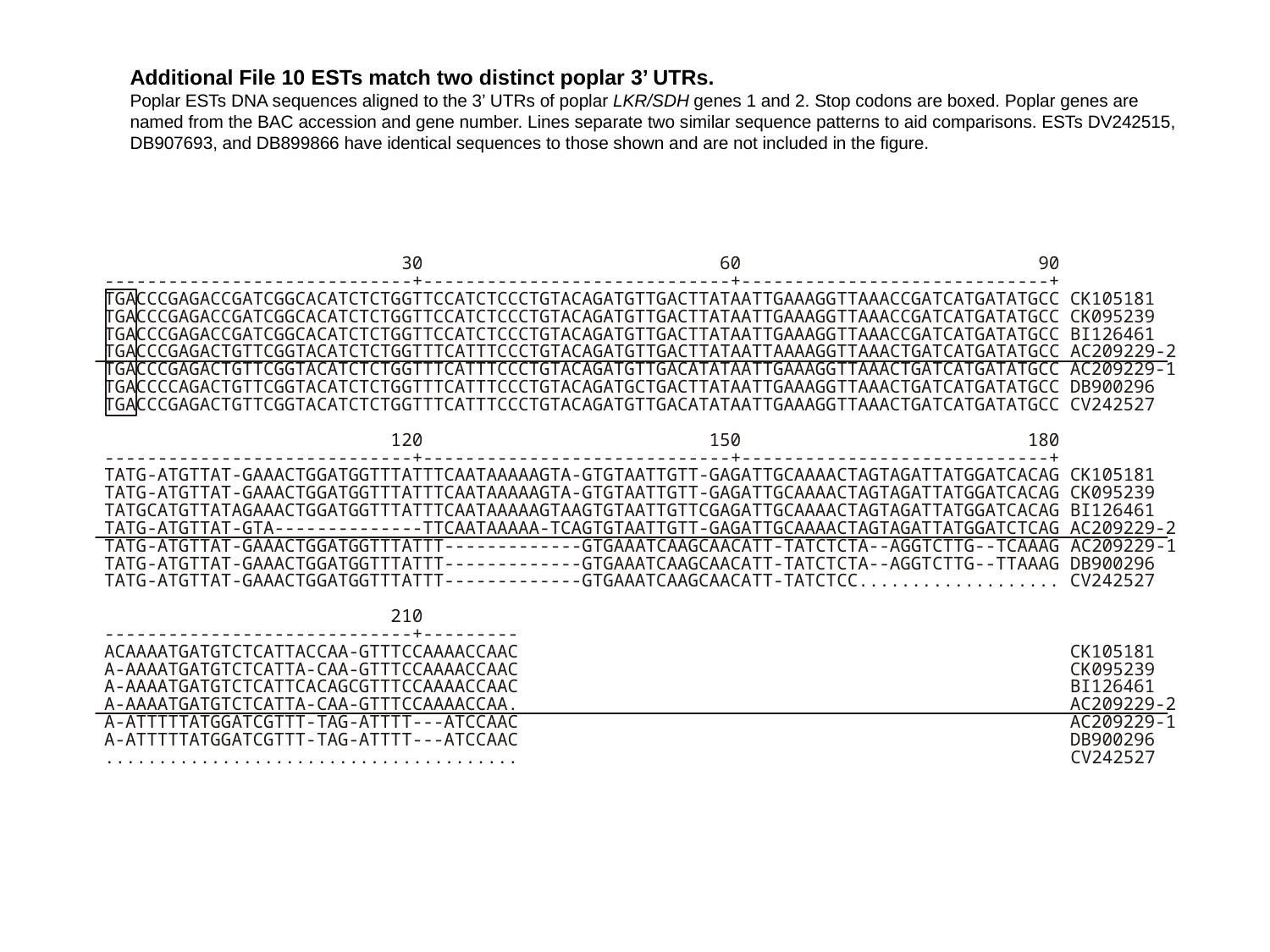

Additional File 10 ESTs match two distinct poplar 3’ UTRs.
Poplar ESTs DNA sequences aligned to the 3’ UTRs of poplar LKR/SDH genes 1 and 2. Stop codons are boxed. Poplar genes are
named from the BAC accession and gene number. Lines separate two similar sequence patterns to aid comparisons. ESTs DV242515,
DB907693, and DB899866 have identical sequences to those shown and are not included in the figure.
