# Supplementary material for: Structural and transcriptional analysis of plant genes encoding the bifunctional lysine ketoglutarate reductase saccharopine dehydrogenase enzyme
Source: BMC Plant Biol. 2010 Jun 16;10:113. doi: 10.1186/1471-2229-10-113 (PMC3017810; doi:10.1186/1471-2229-10-113)
Supplement: Additional File 11 — Monofunctional Brachypodium SDH ESTs. Brachypodium ESTs are aligned to Brachypodium and wheat LKR/SDH exon and intron sequences. [file 1471-2229-10-113-S11.PPT]

## Slide 1
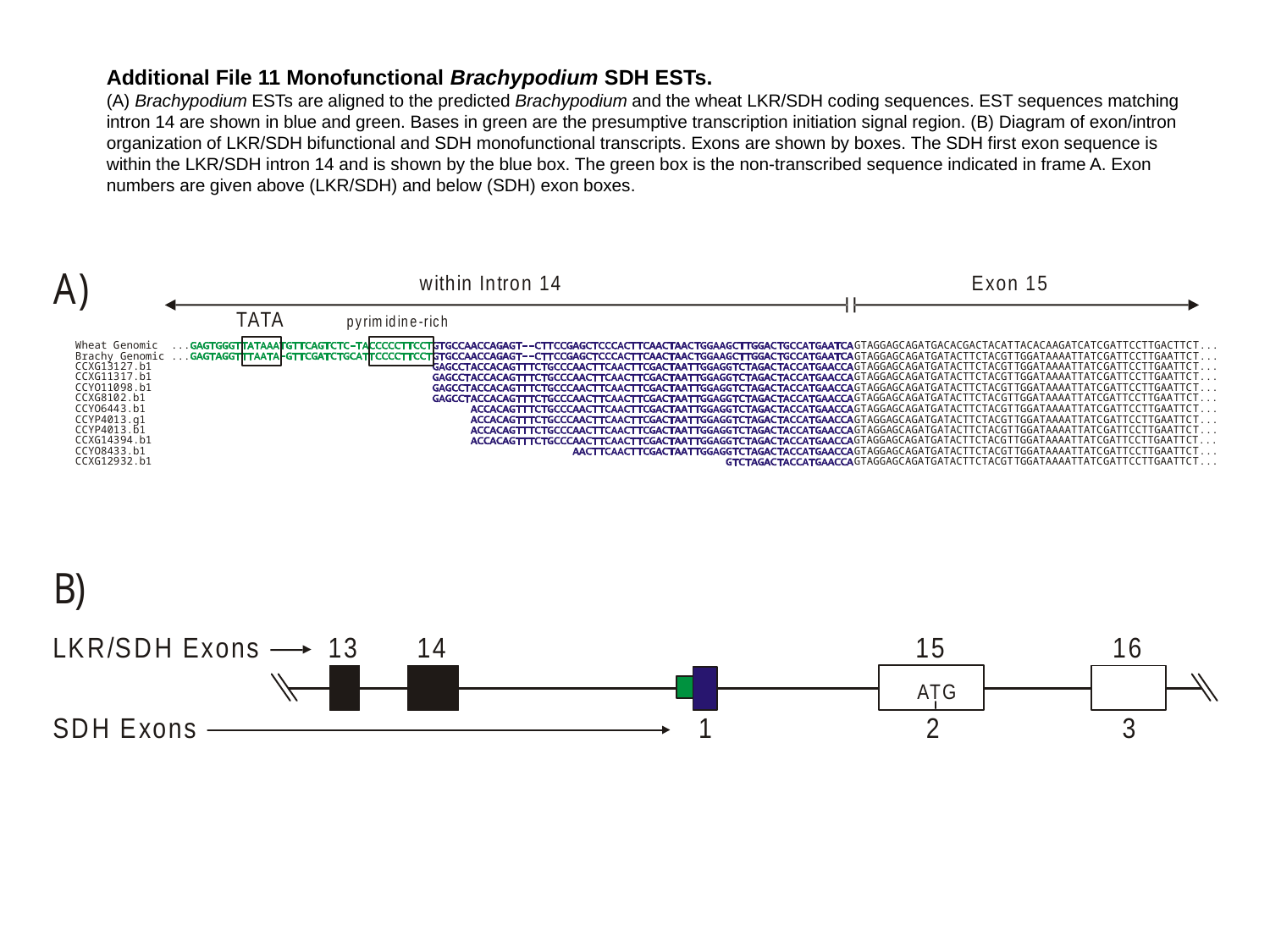

Additional File 11 Monofunctional Brachypodium SDH ESTs.
(A) Brachypodium ESTs are aligned to the predicted Brachypodium and the wheat LKR/SDH coding sequences. EST sequences matching
intron 14 are shown in blue and green. Bases in green are the presumptive transcription initiation signal region. (B) Diagram of exon/intron
organization of LKR/SDH bifunctional and SDH monofunctional transcripts. Exons are shown by boxes. The SDH first exon sequence is
within the LKR/SDH intron 14 and is shown by the blue box. The green box is the non-transcribed sequence indicated in frame A. Exon
numbers are given above (LKR/SDH) and below (SDH) exon boxes.
